# Rational design of asymmetric red fluorescent probes for live cell imaging with high AIE effects and large two-photon absorption cross sections using tunable terminal groups†

**DOI:** 10.1039/c5sc04920b

**Published:** 2016-03-18

**Authors:** Zheng-Feng Chang, Ling-Min Jing, Bin Chen, Mengshi Zhang, Xiaolei Cai, Jun-Jie Liu, Yan-Chun Ye, Xiaoding Lou, Zujin Zhao, Bin Liu, Jin-Liang Wang, Ben Zhong Tang

**Affiliations:** a Beijing Key Laboratory of Photoelectronic/Electrophotonic Conversion Materials, School of Chemistry, Beijing Institute of Technology Beijing China jinlwang@bit.edu.cn; b State Key Laboratory of Luminescent Materials and Devices, South China University of Technology Guangzhou China mszjzhao@scut.edu.cn; c School of Chemistry and Chemical Engineering, Huazhong University of Science and Technology Wuhan China louxiaoding@hust.edu.cn; d Department of Chemical and Biomolecular Engineering, National University of Singapore Singapore 117585

## Abstract

In this work, we report the synthesis of a family of donor–acceptor (D–A) π-conjugated aggregation-induced red emission materials (TPABT, DTPABT, TPEBT and DTPEBT) with the same core 2,2-(2,2-diphenylethene-1,1-diyl)dithiophene (DPDT) and different amounts and different strengths of electron-donating terminal moieties. Interestingly, TPABT and TPEBT, which have asymmetric structures, give obviously higher solid fluorescence quantum efficiencies in comparison with those of the corresponding symmetric structures, DTPABT and DTPEBT, respectively. In particular, the thin film of TPEBT exhibited the highest fluorescence quantum efficiency of *ca.* 38% with the highest *α*_AIE_. Moreover, TPEBT and DTPEBT with TPE groups showed two-photon absorption cross-sections of (*δ*) 1.75 × 10^3^ GM and 1.94 × 10^3^ GM at 780 nm, respectively, which are obviously higher than the other two red fluorescent materials with triphenylamine groups. Then, the one-photon and two-photon fluorescence imaging of MCF-7 breast cancer cells and Hela cells, and cytotoxicity experiments, were carried out with these red fluorescent materials. Intense intracellular red fluorescence was observed for all the molecules using one-photon excitation and for TPABT using two-photon excitation in the cell cytoplasm. Finally, TPEBT is biocompatible and functions well in mouse brain blood vascular visualization. It is indicated that these materials can be used as a specific stain fluorescent probe for live cell imaging.

## Abstract 2

Donor–acceptor π-conjugated aggregation-induced red emission materials for live cell imaging.

## Graphical abstract

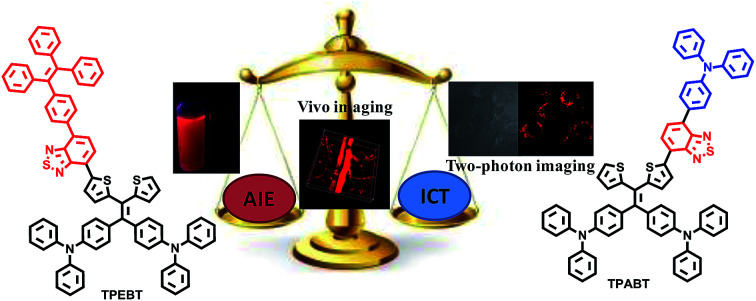

## Introduction

In view of the excellent photophysical properties, and the thermal and chemical stability, fluorescent materials have been applied to bioimaging,^1^ chemosensing^2^ and organic light-emitting diodes (OLEDs).^3^ However, compared to the bright emission in the solution state, the luminescence of most fluorescent materials declined obviously in the solid state due to notorious aggregation-caused quenching (ACQ).^4^ In 2001, the novel phenomenon of aggregation-induced emission (AIE) was first found by Tang's group, in which the emission was very weak in solution but became intense in the aggregated state.^5^ This important finding has become a new method to tackle the ACQ of conventional chromophores and has shown significant academic value and promising applications in cell imaging,^6^ fluorescent sensors^7^ and bioprobe materials.^8^ However, traditional AIE luminophores, such as silole,^9^ tetraphenylethene (TPE)^10^ and tetra(naphthalen-2-yl)ethene (TNE),^11^ generally emit shorter wavelengths. Relatively, among reported AIE materials, organic fluorescent probe materials with excellent red emission and excellent specific staining for cell imaging are still rather limited.^12^ Moreover, most one-photon absorption fluorescent probes are generally excited *via* UV or visible light, which limits their applications due to cell damage and the photobleaching phenomenon.

Compared with traditional one-photon fluorescence imaging, two-photon fluorescence imaging has the advantage of generating high-energy visible fluorescence from low-energy irradiation in the near-infrared region, which draws great interest in the field of materials science due to the various applications including biomedicine, biology and clinical detection.^13^ Therefore, a good two-photon fluorescent probe with a high fluorescence quantum yield in the solid state and a large two-photon absorption (2PA) cross-section is highly desired.^14^ Although intramolecular charge transfer (ICT) could afford large 2PA cross-sections, most 2PA chromophores exhibit weak fluorescence emission in the solid state, suffering from the ACQ effect and strong donor–acceptor interactions.^15^ Recently, some novel symmetric 2PA chromophores were reported which exhibited both large two-photon activities and high brilliant emissions in the solid state by incorporating AIE units.^16^ However, whether the spatial symmetry of 2PA chromophores could affect the application of two-photon fluorescence imaging is lacking systematic and detailed study. Therefore, to enrich the pool of excellent two-photon fluorescent probe materials, it is of great significance to synthesize novel red emissive AIE materials with an in-depth understanding of how to combine large 2PA cross-sections and high fluorescence quantum yields in the solid state at the same time.

**Chart 1:**
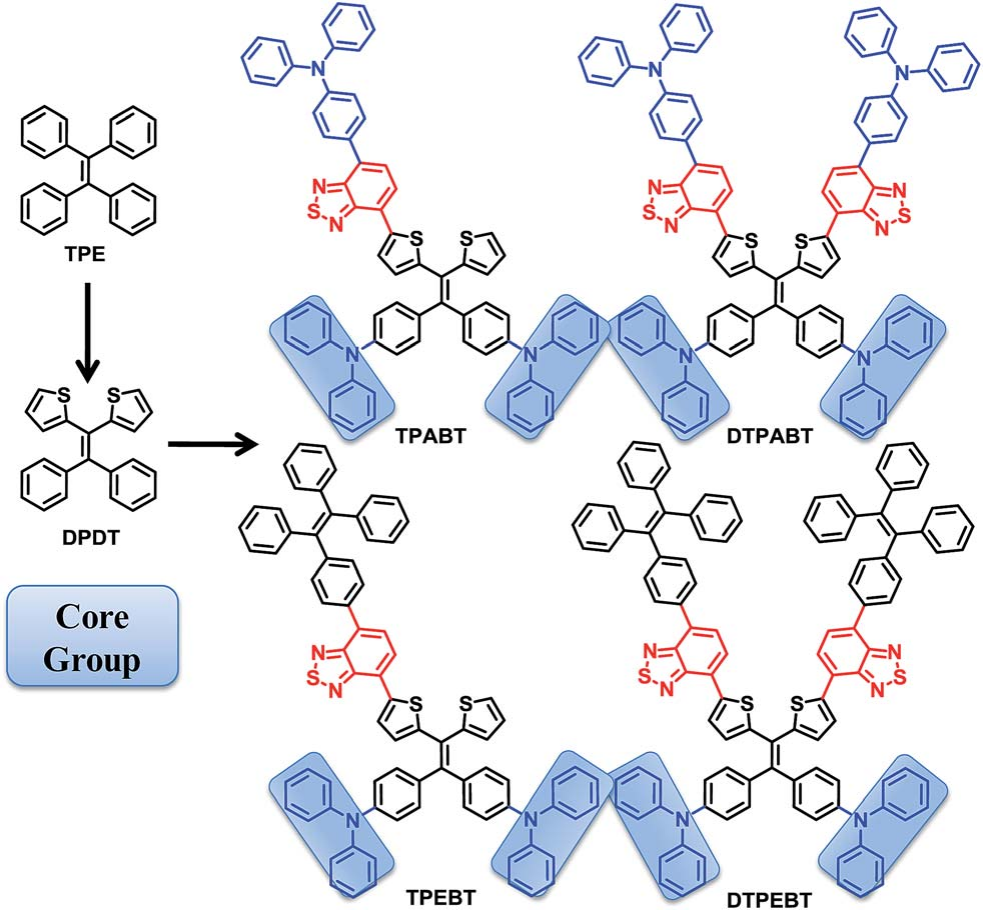
Chemical structures of TPABT, DTPABT, TPEBT, and DTPEBT.

In this work, we design and synthesize a family of donor–acceptor (D–A) π-conjugated aggregation-induced red emission materials (TPABT, DTPABT, TPEBT and DTPEBT) with different spatial symmetries and different strengths of electron-donating terminal moieties (Chart 1). They have the same diphenylamine terminal group and the same four branched core, 2,2-(2,2-diphenylethene-1,1-diyl)dithiophene (DPDT), which showed stronger electron-donating ability and easier modification than the TPE unit.^17^ To modulate the donor–acceptor interaction and get red emission, TPE or triphenylamine mono-functionalized benzo[*c*][1,2,5]thiadiazole (BT) was employed as the other terminal group. Interestingly, TPABT and TPEBT, which have asymmetric structures, give obviously higher solid fluorescence quantum efficiencies in comparison with those of the corresponding symmetric structures, DTPABT and DTPEBT, respectively. Moreover, TPEBT and DTPEBT with TPE groups showed very large two-photon absorption cross-sections of (*δ*) 1.75 × 10^3^ GM and 1.94 × 10^3^ GM at 780 nm, respectively, which are obviously higher than the other two red fluorescent materials with triphenylamine groups. The one-photon and two-photon fluorescence imaging of MCF-7 breast cancer cells and Hela cells, and cytotoxicity experiments were carried out with these red fluorescent probes. Intense intracellular red fluorescence was observed for all the molecules by one-photon excitation and for TPABT by two-photon excitation in the cell cytoplasm. Finally, the *in vivo* imaging experiments of TPEBT are demonstrated in blood vascular imaging in the brain of a living mouse.

## Results and discussion

**Scheme 1:**
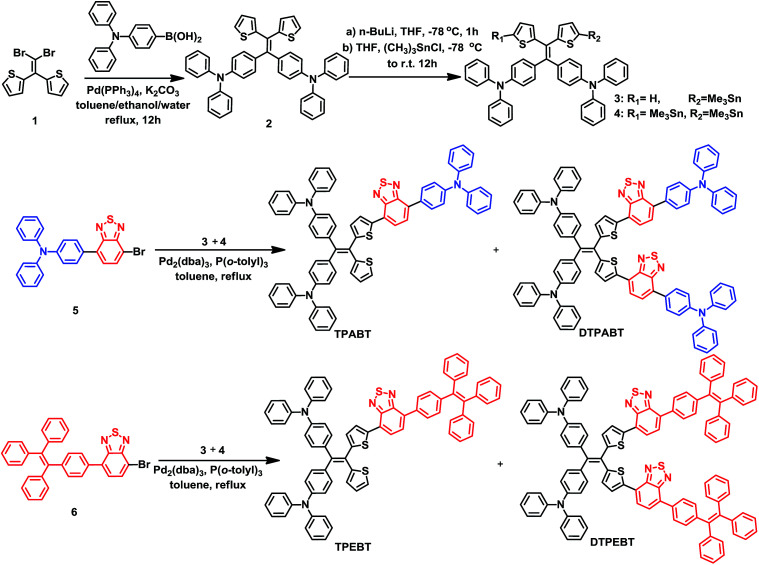
Synthesis of the red AIE molecules TPABT, DTPABT, TPEBT, and DTPEBT.

**Fig. 1:**
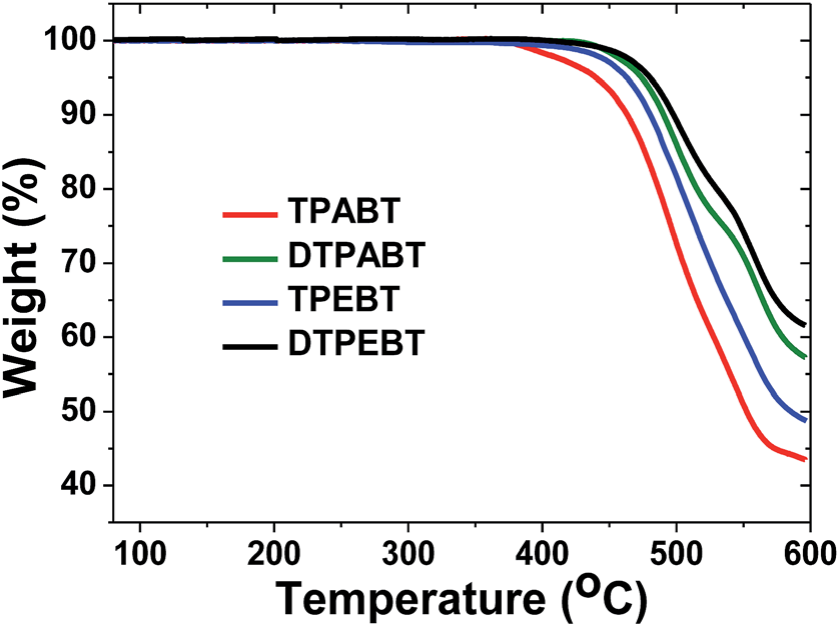
Thermogravimetric analysis (TGA) of TPABT, DTPABT, TPEBT, and DTPEBT with a heating rate of 10 °C min^−1^ under a N_2_ atmosphere.

The synthetic routes to the red AIE materials of TPABT, DTPABT, TPEBT and DTPEBT are presented Scheme 1. The dibromide intermediate 1 was prepared through the Corey–Fuchs reaction. Then 2 was obtained through the Suzuki coupling reaction between the dibromide 1 and (4-(diphenylamino)phenyl)boronic acid as a yellow solid in 65% isolated yield. Then 2 was lithiated by *n*-BuLi followed by quenching with trimethyltin chloride to afford a mixture of monotin 3 and ditin reagents 4 that was directly used in the next step without any further purification. 5 was prepared through the Suzuki reaction between (4-(diphenylamino)phenyl)boronic acid and 4,7-dibromobenzo[*c*][1,2,5]thiadiazole as an orange solid.^18^ Similarly, 6 was synthesized from (4-(1,2,2-triphenylvinyl)phenyl)boronic acid and 4,7-dibromobenzo[*c*][1,2,5]thiadiazole as a yellow solid.^19^ Finally, TPABT and DTPABT were obtained through a Stille coupling reaction between 5 and the mixture of 3 and 4 as a red solid in 45% and 42% isolated yields, respectively. Similarly, TPEBT and DTPEBT were achieved from 6 and the mixture of 3 and 4 as a red solid in 28% and 59% isolated yields, respectively. All compounds were purified by silica gel column chromatography, and their structures and purity were verified by ^1^H NMR, ^13^C NMR and HR-ESI-MS. These materials both exhibit good solubility in common organic solvents such as CHCl_3_, THF, and toluene owing to their branched scaffold. The thermal properties of the four molecules were investigated by thermogravimetric analysis (TGA) (Fig. 1). Under a N_2_ atmosphere, the onset temperature with 5% weight-loss by TGA was over 440 °C for all the red AIE materials, which demonstrates that the thermal stabilities of these molecules are stable enough for photoelectric device application.

**Fig. 2:**
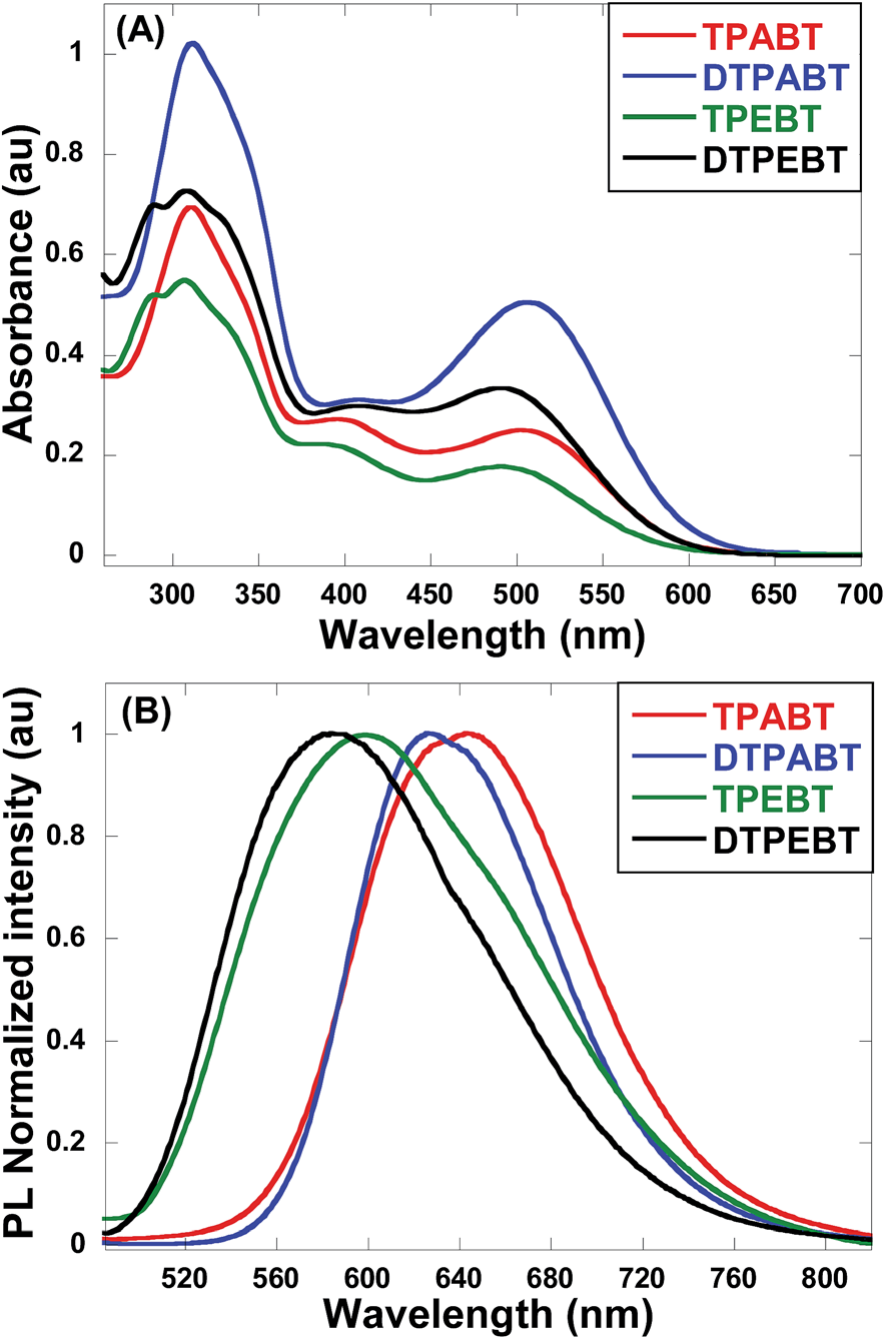
The absorption (A) and emission (B) spectra of the red AIE molecules TPABT, DTPABT, TPEBT, and DTPEBT in THF solutions (10^−5^ M).

The absorption and emission spectra of these red AIE molecules in diluted THF solution were recorded, and the absorption spectra are shown in Fig. 2A. All the molecules show two distinct absorption bands (band I: 270–430 nm; band II: 440–600 nm) in solution due to the π–π* transition of the conjugated backbone for band I and the intramolecular charge transfer (ICT) between the molecular donor and acceptor units for band II. In other words, these red AIE molecules are typical donor–acceptor (D–A) systems. More interestingly, TPABT displayed *λ*_max_ at 502 nm along with an obvious increase in absorbance intensity, which showed a red-shift of 12 nm in comparison with TPEBT due to the attachment of the stronger donor terminal group (TPA). This kind of situation also appears for DTPABT and DTPEBT. Then, as shown in Fig. 2B and Table 1, the emission maxima for TPABT and DTPABT are located at 643 nm and 626 nm, respectively. Compared with that of TPABT, the blue-shifted maximum emission peak of DTPABT is possibly due to steric effects. In addition, the emission peaks of TPABT and TPEBT in solution are located at 643 and 600 nm, respectively. These results indicate that most of the molecules have great promising potential as high performance red fluorescent materials.

**Table 1:** Photophysical properties of TPABT, DTPABT, TPEBT, and DTPEBT in THF solutions^a^ and in thin films^b^

Compound	*λ*_max_ abs.^a^ (sol) (nm)	*λ*_max_ em.^b^ (film) (nm)	*Φ*_F,s_^a^	*Φ*_F,f_^b^
TPABT	310 502	643	9.3%	10.1%
DTPABT	310 508	626	4.0%	4.2%
TPEBT	306 490	600	1.8%	38.2%
DTPEBT	309 491	583	1.3%	2.3%

**Fig. 3:**
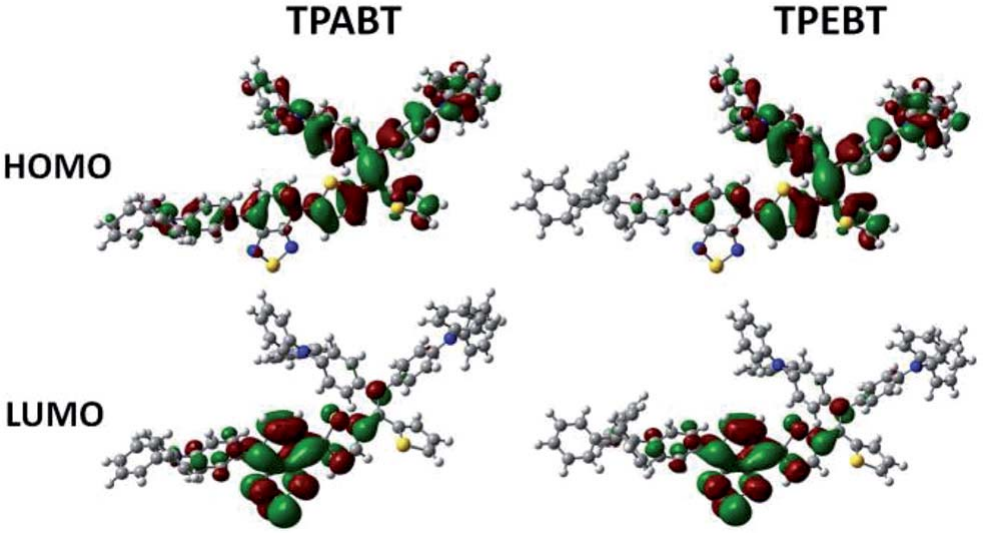
Comparison of the HOMO and LUMO orbital surfaces of TPABT and TPEBT using the DFT B3LYP/6-31G(d) method.

The respective frontier orbital distributions for these red AIE structures based on the DFT B3LYP/6-31G(d) method in the gas phase are presented in Fig. 3 and S1.† The LUMOs of all the AIE structures showed a strong contribution at the electron accepting aromatic moieties (benzothiadiazole). The HOMOs are well delocalized along the whole backbone for TPABT and DTPABT. In contrast for TPEBT and DTPEBT, a slightly stronger localization of the HOMOs around the central core and the diphenylamine terminal group is observed. Moreover, DTPABT presents higher-lying calculated HOMO and LUMO levels and slightly lower bandgaps compared to those of DTPEBT due to the relatively stronger ICT effect of DTPABT (see Table S1†). These results are consistent with the obvious red-shift of the emission peak of DTPABT in comparison to that of DTPEBT.

To quantitatively evaluate the AIE effect of the fluorescent materials, the emission spectra and fluorescent quantum efficiencies (*Φ*_F_) of the thin films and in THF solutions were measured using an integrating sphere method. The *Φ*_F,f_ values were determined to be 10.1%, 4.2%, 38.2% and 2.3% for the TPABT, DTPABT, TPEBT and DTPEBT thin films. We also investigated the *Φ*_F,s_ of TPABT, DTPABT, TPEBT and DTPEBT in THF, the values were determined to be 9.3%, 4.0%, 1.8% and 1.3%, respectively. It is interesting to note that TPEBT with an asymmetric structure and TPE as a terminal group exhibited the highest *Φ*_F,f_ of 38.2%, which might be related to the introduction of TPE hindering the molecular internal rotation. However, a sharply low *Φ*_F,f_ (2.3%) was measured for the film of DTPEBT. Compared with the similar fluorescent quantum efficiencies of TPEBT and DTPEBT in solution, the much lower fluorescent quantum efficiency for the DTPEBT thin film is probably associated with the symmetric conformation of DTPEBT, which leads to much more efficient interchain interactions in the solid state. The AIE-effect is defined as *α*_AIE_ = *Φ*_F,f_/*Φ*_F,s_ and can be used to evaluate the emission contrast ratio between the solid state and solution state. We should note that TPEBT showed the biggest *α*_AIE_ of 21. Meanwhile, TPABT and DTPABT with TPA terminal groups exhibited higher fluorescent quantum efficiencies than that of the other two materials in solution, but they showed a negligible AIE-effect from solution to solid state. These results indicate that spatial symmetry and the introduction of TPE or TPA groups have a significant influence on the fluorescence properties in the solution and solid states.

**Fig. 4:**
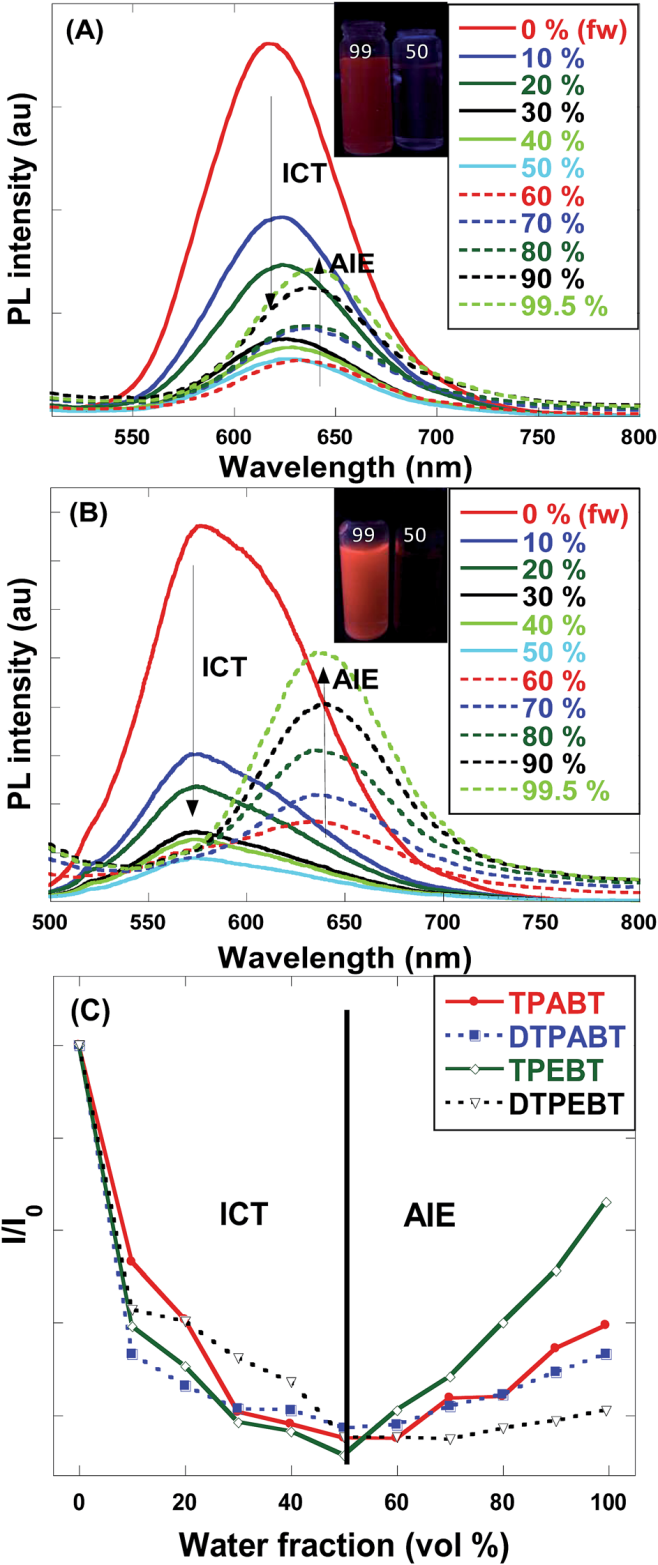
(A) Emission spectra of TPABT in THF–water mixtures with different water fractions (*f*_w_). Inset: photos of TPABT with different water fractions under UV lamp illumination. (B) Emission spectra of TPEBT in THF–water mixtures with different water fractions (*f*_w_). Inset: photos of TPEBT with different water fractions under UV lamp illumination. (C) Changes in the relative PL intensities of TPABT, DTPABT, TPEBT and DTPEBT in THF/water mixtures with different water fractions, where *I*_0_ is the emission intensity in pure THF solution.

For an in-depth understanding of the AIE effect, we measured the emission spectra of the four molecules in THF and THF–water mixtures with different water fractions (*f*_w_) to study the emission change (Fig. 4 and S2†). To our surprise, the emission changes for these four molecules were similar. For example, TPEBT showed nearly sustained decrease until the *f*_w_ reached 60%, accompanied by a red-shifting of the emission peaks by about 60 nm. This phenomenon could be reasonably explained by an intramolecular charge-transfer (ICT) mechanism. Once the *f*_w_ was increased beyond 60%, there was obviously enhanced emission at about 630 nm, demonstrating AIE activity. In this stage, TPEBT began to aggregate because the solvating power of the aqueous mixture decreased, making the emission enhanced due to the restriction of intramolecular rotation (RIR) effect. Meanwhile, the ICT effect is efficiently weakened. TPABT showed similar emission spectra to that of TPEBT. However, the emission band of TPABT in the solvent mixture with *f*_w_ = 60% was red-shifted only about 20 nm, compared with that in pure THF solution. That may be because TPABT has weaker ICT. It's easy to see the AIE effect in Fig. 4C. The AIE behavior of TPEBT is best, due to the introduction of the strong AIE luminophore (TPE). Interestingly, the AIE effects of TPABT and DTPABT seem to be weak with the introduction of TPA moieties because more TPA groups might generate a larger steric hindrance, in which the rotation of the molecules could not be restricted efficiently after aggregation. To our surprise, DTPEBT has the worst AIE property. That may be the joint effect of symmetric conformation and stronger intramolecular charge transfer. This trend looks to be the same as the absolute quantum yield (*Φ*_F_) measurements.

**Fig. 5:**
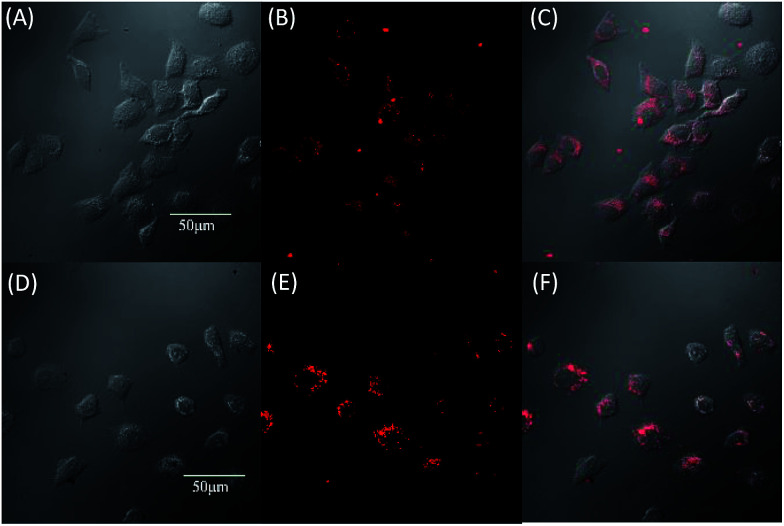
Fluorescence images of MCF-7 by DTPEBT (A–C) and TPABT (D–F). (A) and (D) are bright field image cells, (B) and (E) are fluorescence images, (C) and (F) are merged images, incubated for 3 hours with 20 μM.

**Fig. 6:**
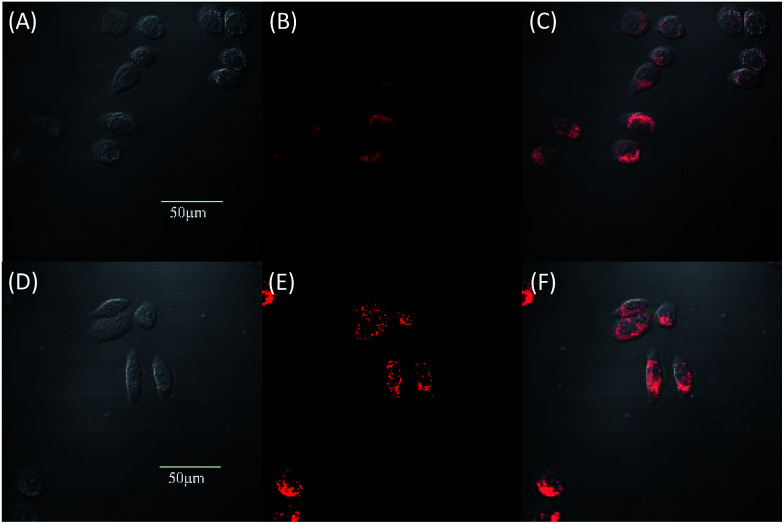
Fluorescence images of Hela by DTPEBT (A–C) and TPABT (D–F). (A) and (D) are bright field image cells, (B) and (E) are fluorescence images, (C) and (F) are merged images, incubated for 3 hours with 20 μM.

Due to the strong red fluorescence of these materials, they are used as one-photon fluorescent probes for live cell imaging. Human breast cancer cells (MCF-7 cells) and Hela cells were chosen as the model cell-lines for the fluorescence imaging study by confocal laser scanning microscopy (CLSM). The cells were incubated for 3 h in a culture medium containing 20 μM of these molecules, and then were used for one-photon confocal imaging. Fig. 5, 6, S3 and S4† show the images of the cells, taken under 405 nm with a 560–660 nm band pass filter. Intense intracellular red fluorescence was observed from all the molecules in the cell cytoplasm. This indicates that these molecules can be used as a specific stain for cell imaging.^20^

**Fig. 7:**
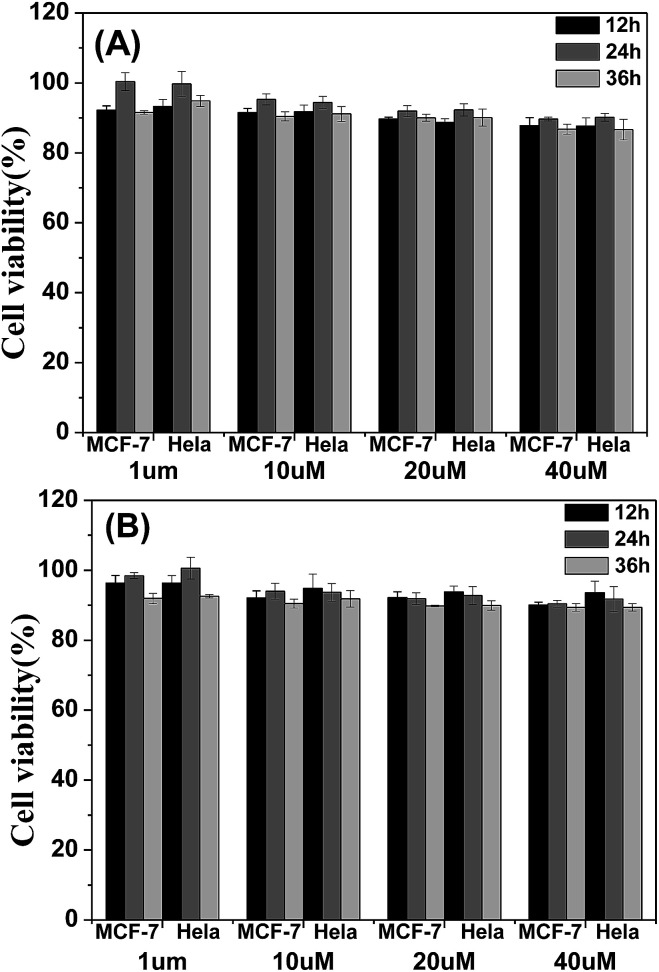
Metabolic viability of MCF-7 breast cancer cells and Hela cells after incubation with red emission AIE molecules (A) TPABT and (B) TPEBT for 12, 24 and 36 h at different concentrations.

The cytotoxicity of the four materials was evaluated through the investigation of the metabolic viability of human breast cancer cells (MCF-7 cells) and Hela cells in Fig. 7 and S5.† The result turns out to be satisfying, showing that all the materials are nontoxic as the stained cells are in good health conditions. At last the cell viability remains above 85% within 36 h and 40 μM under the experimental conditions, indicating the low cytotoxicity of the molecules. Therefore, these AIE molecules are expected to be promising candidates for cell imaging with some advantages such as AIE characteristics, intense red emission and excellent biocompatibility.

**Fig. 8:**
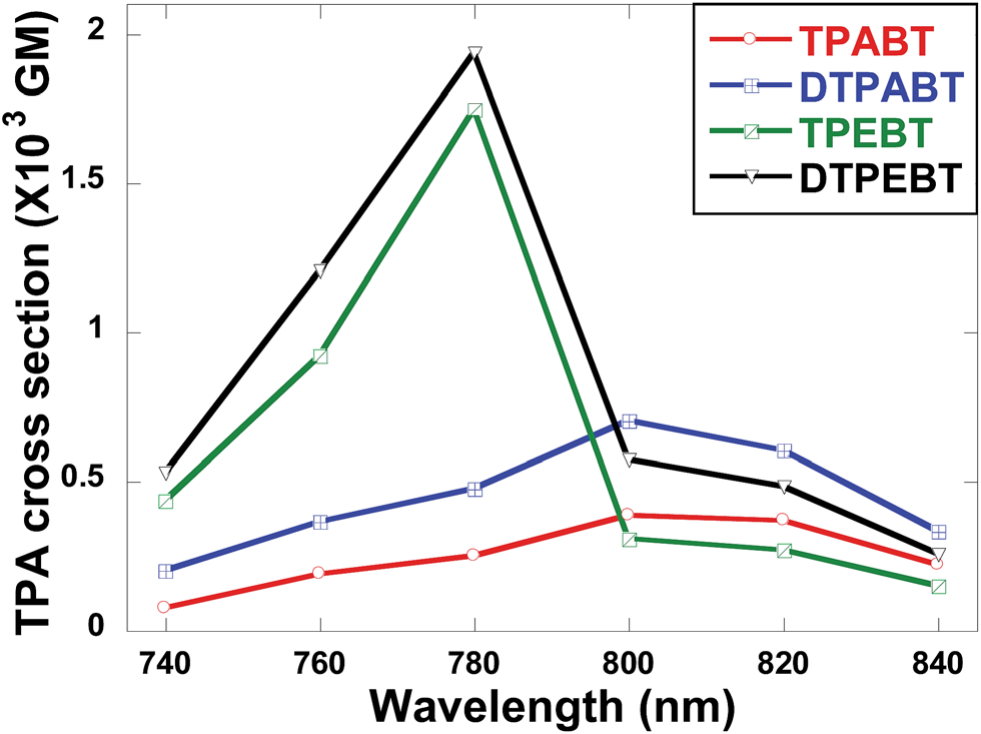
Two-photon absorption cross-sections of the red emission AIE molecules TPABT, DTPABT, TPEBT, and DTPEBT.

The two-photon absorption (2PA) spectra of the molecules were studied using a two-photon-excited fluorescence (TPEF) technique with a femtosecond pulsed laser source. According to the laser availability, the spectra were collected in the 740 nm to 840 nm range at 20 nm intervals. The relative TPEF intensities of the molecules were measured using 2′,7′-dichlorofluorescein in THF as the standard. The 2PA cross-sections (*δ*) of the molecules were measured in the wavelength range from 740–840 nm, as shown in Fig. 8. The maximum *δ* is 0.39 × 10^3^ GM at 800 nm for TPABT, 0.70 × 10^3^ GM at 800 nm for DTPABT, 1.75 × 10^3^ GM at 780 nm for TPEBT and 1.94 × 10^3^ GM at 780 nm for DTPEBT. Notably, DTPABT and DTPEBT with their symmetric conformations showed obviously larger 2PA cross-sections than those of TPABT and TPEBT. Such results indicate that an extended π-system could enhance the 2PA cross-section of a whole molecule. Moreover, very interestingly, the 2PA cross-section of DTPEBT is much enhanced in comparison with that of DTPABT due to the extended π-conjugated length from the double bonds. Such results indicate that incorporating TPE as a terminal group is an effective approach for enhancing the 2PA cross-section of a molecule.^21^ Fig. S6† shows a comparison of the one-photon excited fluorescence (OPEF) spectra with the TPEF spectra of TPABT, DTPABT, TPEBT and DTPEBT excited at 430 and 780 nm in THF solution at 10^−5^ M. The TPEF spectra resembles the OPEF spectra except for a red-shift, suggesting a volume reabsorption effect of the fluorescence with the solution, and different spectral selection rules.^22^ Overall, the 2PA cross-sections of our red emission AIE materials are impressive and can be modulated by different spatial symmetries and the strength of the electron-donating terminal moieties, which encourages us to further explore their potential application in two-photon fluorescence imaging.

**Fig. 9:**
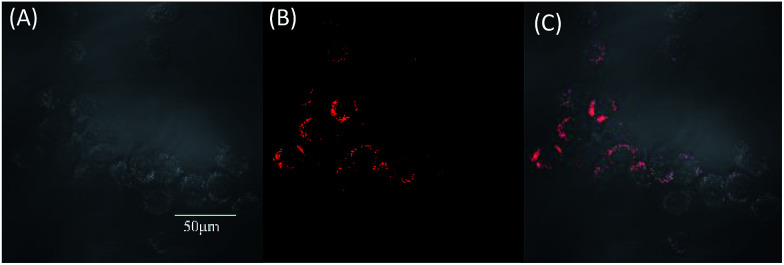
Two-photon excited fluorescence imaging of human breast cancer cells (MCF-7 cells) after 3 h incubation with TPABT at 37 °C. The images were recorded upon 980 nm excitation with a 560–660 nm band pass filter. (A) Brightfield image cells, (B) two-photon excited fluorescence, (C) two-photon excited fluorescence/brightfield overlay. The scale bar is 50 μm.

To evaluate the effect of these molecules in living cells, two-photon fluorescence microscopy images were obtained from MCF-7 incubated with TPABT and TPEBT, owing to their lower cytotoxicity toward live cells and two-photon behavior, together with the higher fluorescence quantum efficiencies. To minimize the side effects of the organic solvent toward live cells, each molecule was dissolved in DMSO. To our surprise, the two-photon excited fluorescence images (Fig. 9 and S7†) of the TPABT live cells were successfully taken and clearly displayed the cytoplasm structure. Nevertheless, the TPEBT live cell images were unsuccessful and seldom displayed the live cells, owing to the weak 2PA of TPEBT at such long wavelengths.^23^ In view of this, the red-fluorescent TPABT molecule is more advantageous than TPEBT for two-photon excited fluorescence imaging.

**Fig. 10:**
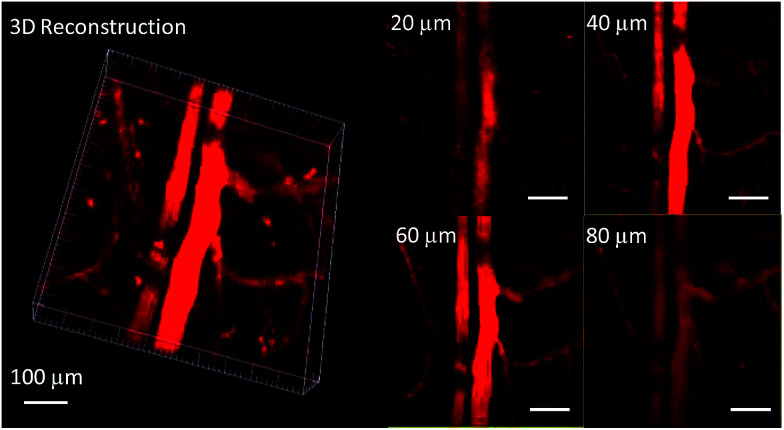
Two-photon fluorescence images of ear blood vessels stained with TPEBT NPs including 3D reconstructed images of blood vessels when the TPEBT NPs were excited at 780 nm, and images at different vertical depths of the mouse ear. All the images share the same scale bar of 100 μm.

In order to study the effect of these molecules in an animal model, two-photon fluorescence imaging microscopy was further discussed for real-time *in vivo* two-photon fluorescence imaging with TPEBT nanoparticles (NPs) owing to its best two-photon behavior. *In vivo* imaging of the blood vasculature of a mouse ear was conducted using the NPs as the blood vessel visualizing agent. The TPEBT NPs were excited at 780 and 980 nm and emitted signals that were collected at 542 ± 27 nm. Fig. 10 and S8† show the 3D reconstructions and the distinct blood vasculature at different depths. After injection of the NPs, it is obvious that the blood vascular network including the major blood vasculature, small capillaries, and even arteries located deeply over 80 μm could be clearly observed in blood vessels with red fluorescence. The high-resolution, 3D reconstructed image further illustrates the applicability of the TPEBT NPs for TPEF *in vivo* imaging. This indicates the excellent stability and biocompatibility of these molecules in a living biological system.

## Conclusions

In summary, a series of donor–acceptor (D–A) π-conjugated red fluorescent materials (TPABT, DTPABT, TPEBT and DTPEBT) with the same branched core and different spatial symmetries and strengths of electron-donating terminal moieties (TPE or TPA) was synthesized and characterized. The thin film of TPEBT with an asymmetric structure and TPE as a terminal group exhibited the highest fluorescence quantum efficiency of 38.2% with the highest *α*_AIE_. Moreover, our molecule design strategy means that the two compounds with TPE as a terminal group have very large two-photon absorption cross sections in comparison with the two red emission materials with triphenylamine groups. What's more, our investigation demonstrates that these molecules act as one-photon and two-photon fluorescent probes and can be successfully applied for the fluorescence imaging of MCF-7 breast cancer cells and Hela cells, and in the corresponding cytotoxicity experiments of live cells imaging. Notably, when TPABT was used as one-photon and two-photon fluorescent probes, intense intracellular red fluorescence was observed in the cell cytoplasm. Red emissive biocompatible TPEBT was applied in blood vascular imaging of a mouse ear, demonstrating its great potential as a two-photon excited contrast agent in biological systems. It is indicated that such asymmetric and TPA terminal group materials can be used as a specific stain fluorescent probe for live cell imaging. This study provides fundamental structure design guidelines for further novel red AIE molecules with large 2PA cross-sections as promising candidates for live-cell imaging in clinical trials.

## Experimental section

### General procedures

All air and water sensitive reactions were performed under a nitrogen atmosphere. Tetrahydrofuran and toluene were dried over Na/benzophenone ketyl and were freshly distilled prior to use. The other materials were of the common commercial level and were used as received. Thin layer chromatography (TLC) was conducted on flexible sheets precoated with SiO_2_ and the separated products were visualized by UV light. Column chromatography was conducted using SiO_2_ (300 mesh) from Fisher Scientific. ^1^H and ^13^C NMR spectra were recorded on a Bruker ARX-400 (400 MHz) or ARX-500 (500 MHz) spectrometer, using CDCl_3_. All chemical shifts were reported in parts per million (ppm). The ^1^H NMR chemical shifts were referenced to TMS (0 ppm), and the ^13^C NMR chemical shifts were referenced to CDCl_3_ (77.23 ppm). HR-ESI-MS data were recorded on a Bruker APEX IV mass spectrometer. Thermal gravimetric analysis (TGA) was carried out on a TA Instrument Q500 analyzer. Absorption spectra were recorded on a PerkinElmer Lambda 750 UV-vis spectrometer. Photoluminescence was recorded on a Perkin-Elmer LS 55 spectrofluorometer and a HORIBA JobinYvon Nanolog FL3-2iHR spectrometer.

### Cell culture

MCF-7 (human breast cancer cells) cells were obtained from the Institute of Basic Medical Sciences (IBMS) of the Chinese Academy of Medical Sciences (CAMS). All cell lines were maintained under standard culture conditions (atmosphere of 5% CO_2_ and 95% air at 37 °C) in RPMI 1640 medium, supplemented with 10% FBS (fetal calf serum).

### Cell fluorescence imaging

MCF-7 cells were grown in the exponential phase of growth on 35 mm glass-bottom culture dishes (*Φ* 20 mm) for 1–2 days to reach 70–90% confluency. These cells were used in co-localization experimentation. The cells were washed three times with RPMI 1640, and then incubated with 1 mL RPMI 1640 containing red AIE molecules (20 μM) in an atmosphere of 5% CO_2_ and 95% air for 3 h at 37 °C. The cells were washed three times with 1 mL PBS at room temperature, and then 1 mL PBS was added to the culture medium to observe under a confocal microscope (Olympus FV1000). Channel 1: excitation: 405 nm, emission collected: 560–660 nm.

#### Cytotoxicity

The metabolic activities of the MCF-7 breast cancer cells and Hela cells were evaluated using methylthiazolyldiphenyl-tetrazolium (MTT) assays. The cells were seeded in 96-well plates (Costar, IL, USA) at an intensity of 4 × 10^4^ cells mL^−1^. After 24 h incubation, the medium was replaced by the TPABT, DTPABT, TPEBT and DTPEBT suspensions at different concentrations in DMEM containing 10% FBS and 1% penicillin streptomycin, and the cells were then incubated for 12, 24 and 36 h. After the designated time intervals, the wells were washed three times with 1× PBS buffer, and 100 μL of freshly prepared MTT (0.5 mg mL^−1^) solution in culture medium was added into each well. The MTT medium solution was carefully removed after 3 h incubation in the incubator. Dimethyl sulfoxide (DMSO, 150 μL) was then added into each well and the plate was gently shaken for 10 min at room temperature to dissolve all the precipitates formed. The absorbance of MTT at 490 nm was monitored by the microplate reader (Genios Tecan).

### Fabrication of TPEBT NPs

The TPEBT-loaded DSPE-PEG 2000 NPs were prepared through a modified nanoprecipitation method. Briefly, 1 mL of THF solution containing 1 mg of TPEBT and 2 mg of DSPE-PEG 2000 was poured into 9 mL of water. This was followed by sonicating the mixture for 60 s at 10 W output using a microtip probe sonicator (XL2000, Misonix Incorporated, NY). The mixture was then stirred at room temperature overnight to evaporate the THF. The obtained solution was filtered using a 0.20 μm syringe-driven filter to collect the products.

#### Brain blood vascular imaging

The experimental set up for brain imaging is described elsewhere.^24^ The small 2 mm circular piece of parietal bone was excised using a dental drill, exposing the meninges and the brain of the immobilized mouse. For the TPEF experiments, the mice were anesthetized (150 mg kg^−1^ ketamine and 10 mg kg^−1^ xylazine) and placed on a heating pad to maintain a core body temperature of 37 °C throughout each imaging procedure. 200 μL of TPEBT NPs at 50 × 10^−6^ M TPEBT was administered *via* retro-orbital injection prior to imaging. All procedures were performed under the institution's IACUC (Institutional Animal Care and Use Committee) guidelines. A TriM Scope II single-beam two-photon microscope (LaVision BioTec) with a tunable 680–1080 nm laser (coherent) was used to acquire the images. The TPEBT NPs and second harmonic generation were excited at 780 and 980 nm, and the emitted light was split by 520 and 640 nm long pass mirrors and detected through 542/27 nm filters.

#### Synthesis of TPABT and DTPABT

In a 100 mL two-neck round-bottom flask, 4-(7-bromobenzo[*c*][1,2,5]thiadiazol-4-yl)-*N*,*N*-diphenylaniline (0.05 g, 0.12 mmol), 4,4′-(2,2-bis(5-(trimethylstannyl)thiophen-2-yl)ethene-1,1-diyl)bis(*N*,*N*-diphenylaniline) (0.28 g, 0.30 mmol), tris(dibenzylideneacetone)dipalladium(0) (0.005 g, 0.005 mmol) and *o*-tolyl phosphine (0.01 g, 0.02 mmol) were added. The flask was evacuated and back-filled with N_2_ three times, and then degassed toluene (40 mL) was injected into the mixture. The resulting solution was stirred at refluxing temperature for 12 h under the N_2_ atmosphere. After being cooled to room temperature, the solvents were then removed under reduced pressure. The dark residue was purified by silica gel chromatography, eluting with PE–CH_2_Cl_2_ (1 : 1) to give a red solid (0.12 g, 45%, TPABT) and another red solid (0.15 g, 42%, DTPABT).

##### TPABT

^1^H NMR (CDCl_3_, 400 MHz, ppm): *δ* 7.98–7.97 (d, *J* = 4.0 Hz, 1H, Th-H), 7.89–7.87 (d, *J* = 8.4 Hz, 2H, Ph-H), 7.82–7.80 (d, *J* = 7.2 Hz, 1H, Ph-H), 7.69–7.67 (d, *J* = 7.6 Hz, 1H, Ph-H), 7.32–7.27 (m, 6H), 7.25–7.00 (m, 29H), 6.95–6.88 (m, 9H). ^13^C NMR (CDCl_3_, 100 MHz, ppm): *δ* 154.2, 153.0, 148.3, 147.6, 147.1, 146.9, 142.6, 137.2, 137.0, 132.2, 131.9, 130.9, 130.1, 129.6, 129.4, 129.3, 127.4, 127.1, 126.4, 125.6, 125.1, 124.8, 124.6, 123.6, 123.4, 123.2, 123.1, 123.0, 122.6. HR-ESI-MS (*m*/*z*): calcd for C_70_H_49_N_5_S_3_: 1055.3150 (100%). Found: 1056.3207 ([M + H]^+^, 100%).

##### DTPABT

^1^H NMR (CDCl_3_, 400 MHz, ppm): *δ* 8.01–8.00 (d, *J* = 4.0 Hz, 2H, Th-H), 7.89–7.87 (d, *J* = 8.8 Hz, 4H, Ph-H), 7.84–7.82 (d, *J* = 6.8 Hz, 2H, Ph-H), 7.69–7.67 (d, *J* = 7.6 Hz, 2H, Ph-H), 7.31–7.28 (m, 8H), 7.16–7.05 (m, 36H), 6.96–6.92 (m, 10H). ^13^C NMR (CDCl_3_, 125 MHz, ppm): *δ* 154.3, 153.1, 148.4, 147.8, 147.4, 132.3, 131.1, 130.2, 129.6, 129.4, 127.4, 125.2, 124.7, 123.6, 123.3, 123.2, 123.1. HR-ESI-MS (*m*/*z*): calcd for C_94_H_64_N_8_S_4_: 1433.4170 (100%). Found: 1433.4167 (100%).

#### Synthesis of TPEBT and DTPEBT

In a 100 mL two-neck round-bottom flask, 4-bromo-7-(4-(1,2,2-triphenylvinyl)phenyl)benzo[*c*][1,2,5]thiadiazole (0.11 g, 0.10 mmol), 4,4′-(2,2-bis(5-(trimethylstannyl)thiophen-2-yl)ethene-1,1-diyl)bis(*N*,*N*-diphenylaniline) (0.23 g, 0.25 mmol), tris(dibenzylideneacetone)dipalladium(0) (0.005 g, 0.005 mmol) and *o*-tolyl phosphine (0.01 g, 0.02 mmol) were added. The flask was evacuated and back-filled with N_2_ three times, and then degassed toluene (40 mL) was injected into the mixture. The resulting solution was stirred at refluxing temperature for 12 h under the N_2_ atmosphere. After being cooled to room temperature, the solvents were then removed under reduced pressure. The dark residue was purified by silica gel chromatography, eluting with PE–CH_2_Cl_2_ (1 : 1) to give a red solid (0.06 g, 28%, TPEBT) and another red solid (0.19 g, 59%, DTPEBT).

##### TPEBT

^1^H NMR (CDCl_3_, 400 MHz, ppm): *δ* 7.97–7.96 (d, *J* = 3.6 Hz, 1H, Th-H), 7.78–7.86 (m, 3H, Ph-H), 7.68–7.66 (d, *J* = 7.2 Hz, 1H, Ph-H), 7.25–7.00 (m, 41H), 6.95–6.87 (m, 8H). ^13^C NMR (CDCl_3_, 100 MHz, ppm): *δ* 154.1, 153.0, 148.1, 147.7, 147.2, 146.9, 146.0, 144.0, 143.95, 143.85, 142.7, 141.7, 140.7, 139.8, 137.2, 137.0, 135.3, 132.2, 131.8, 131.7, 131.58, 131.57, 131.1, 129.9, 129.4, 129.3, 128.0, 127.9, 127.8, 127.3, 126.8, 126.73, 126.68, 126.6, 126.4, 125.5, 125.3, 124.8, 124.6, 123.3, 123.2, 123.0, 122.6. HR-ESI-MS (*m*/*z*): calcd for C_78_H_54_N_4_S_3_: 1142.3511 (100%). Found: 1143.3505 ([M + H]^+^, 100%).

##### DTPEBT

^1^H NMR (CDCl_3_, 400 MHz, ppm): *δ* 8.01–8.00 (d, *J* = 3.6 Hz, 2H, Th-H), 7.82–7.80 (d, *J* = 7.6 Hz, 2H, Ph-H), 7.78–7.76 (d, *J* = 8.0 Hz, 4H, Ph-H), 7.69–7.67 (d, *J* = 7.6 Hz, 2H, Ph-H), 7.20–7.02 (m, 56H), 6.96–6.91 (m, 8H). ^13^C NMR (CDCl_3_, 100 MHz, ppm): *δ* 154.1, 152.9, 147.7, 147.2, 144.0, 143.96, 143.86, 141.7, 140.7, 135.3, 132.2, 131.9, 131.7, 131.59, 131.57, 129.4, 128.5, 128.1, 128.0, 127.9, 126.8, 126.73, 126.69, 124.7, 123.12, 123.09. HR-ESI-MS (*m*/*z*): calcd for C_110_H_74_N_6_S_4_: 1607.4891 (100%). Found: 1607.4887 (100%).

## Supplementary Material

SC-007-C5SC04920B-s001
