# Supplementary material for: Rational design of asymmetric red fluorescent probes for live cell imaging with high AIE effects and large two-photon absorption cross sections using tunable terminal groups
Source: Chem Sci. 2016 Mar 18;7(7):4527–36. doi: 10.1039/c5sc04920b (PMC6018563; doi:10.1039/c5sc04920b)
Supplement: SC-007-C5SC04920B-s001 [file SC-007-C5SC04920B-s001.pdf]

Electronic Supplementary Material (ESI) for Chemical Science.
This journal is © The Royal Society of Chemistry 2016

Supporting Information

Rational design of asymmetric red fluorescent probes for live cell imaging with high AIE effect and large two-photon absorption cross section by tunable terminal group

Zheng-Feng Chang,[a] Ling-Min Jing,[a] Bin Chen,[b] Mengshi Zhang,[c] Xiaolei Cai,[d] Jun-Jie Liu,[a] Yan-Chun Ye,[a] Xiaoding Lou,*[c] Zujin Zhao,*[b] Bin Liu*[d] Jin-Liang Wang*[a] and Ben Zhong Tang[b]

a) Beijing Key Laboratory of Photoelectronic/Electrophotonic Conversion Materials, Beijing Institute of Technology, Beijing, 100081, China.
E-mail: jinlwang@bit.edu.cn
b) State Key Laboratory of Luminescent Materials and Devices, South China University of Technology, Guangzhou 510640, China.
E-mail: mszjzhao@scut.edu.cn
c) School of Chemistry and Chemical Engineering, Huazhong University of Science and Technology, Wuhan 430074, China.
E-mail: louxiaoding@hust.edu.cn
d) Department of Chemical and Biomolecular Engineering, National University of Singapore, Singapore 117585

1. Experimental Details

1: To a solution of di(thiophen-2-yl)methanone (1.06 g, 5.46 mmol), CBr_4 (3.57 g, 10.93 mmol) and PPh_3 (5.73 g, 21.86 mmol) in toluene (50 mL). After refluxing for 24 h, the mixture was cooled to 25 °C and poured into an aq. NH_4Cl solution. The aqueous layer was extracted with CH_2Cl_2, and then the combined organic phase was washed with brine and dried ($MgSO_4$). The solvents were then removed under reduced pressure. The residue was purified by silica gel chromatography, eluting with petroleum ether (PE) to give yellow oil (1.23 g, 65%). ^1H NMR ($CDCl_3$, 400 MHz, ppm): δ 7.39-7.41 (d, *J* = 5.2 Hz, 2H), 7.10-7.11 (d, *J* = 3.2 Hz, 2H), 7.00-7.02 (t, *J* = 4.4 Hz, 2H). ^{13}C NMR ($CDCl_3$, 100 MHz, ppm): δ 142.1, 134.3, 130.0, 127.6, 126.8, 92.2. HR-EIS-MS(*m/z*): calcd for $C_{10}H_6Br_2S_2$: 349.8257(100%). Found: 350.8330 ($[M+H]^+$, 100%).

2: To a solution of 1 (0.50 g, 1.73 mmol), (4-(diphenylamino)phenyl)boronic acid (0.25 g, 0.72 mmol), $Pd(PPh_3)_4$ (0.07 g, 0.06 mmol) in toluene (40 mL) and ethanol(80 mL), aqueous NaOH solution (1.0 M, 5.0 mL) and were added. After refluxing for 12 h under N_2, the mixture was cooled to 25 °C and poured into an aq. NH_4Cl solution. The aqueous layer was extracted with CH_2Cl_2, and then the combined organic phase was washed with brine and dried ($MgSO_4$). The solvents were then removed under reduced pressure. The residue was purified by silica gel chromatography, eluting with petroleum ether (PE)-CH_2Cl_2 (4:1) to give yellow solid (0.31 g, 64%). ^1H NMR ($CDCl_3$, 400MHz, ppm) δ: 7.22-7.26 (m, 8H), 7.18-7.20 (dd, *J* = 4.8 Hz, 2H), 7.07-7.09 (d, *J* = 7.6 Hz, 8H), 6.98-7.04 (m, 8H), 6.87-6.89 (d, *J* =

8.4 Hz, 4H), 6.83-6.84 (t, *J* = 4.0 Hz, 2H), 6.80-6.81 (dd, *J* = 3.6 Hz, 2H). ^{13}C NMR ($CDCl_3$, 100MHz, ppm) δ: 147.8, 146.8, 146.5, 142.0, 137.2, 1319, 129.6, 129.4, (126.23, 126.18), 125.6, 124.7, 123.1, 122.9. HR-ESI-MS (m/z): calcd for $C_{46}H_{34}N_2S_2$: 678.2163 (100%). Found: 678.2168 (100%).

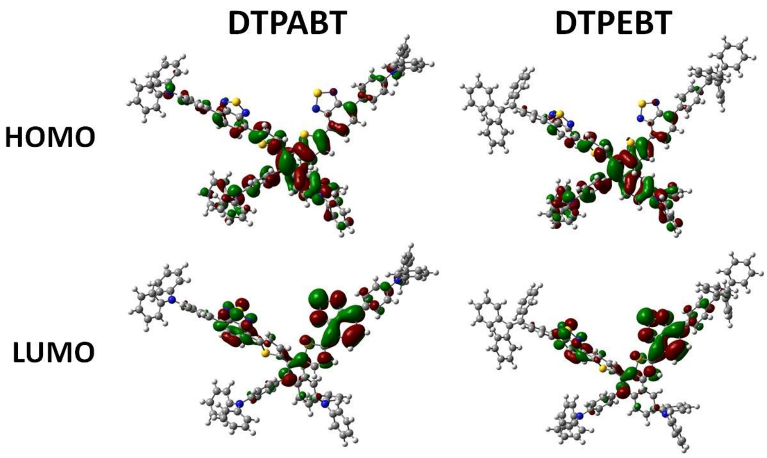

Figure S1.Comparison of HOMO and LUMO orbital surfaces of **DTPABT** and **DTPEBT** using DFT B3LYP/6-31G(d) method.

Table S1. Summary of calculated parameters for these AIE molecules using DFT B3LYP/6-31G(d) method.

Compd	HOMO (eV)	LUMO (eV)
**TPABT**	-4.61	-2.40
**TPEBT**	-4.65	-2.44
**DTPABT**	-4.54	-2.42
**DTPEBT**	-4.60	-2.47

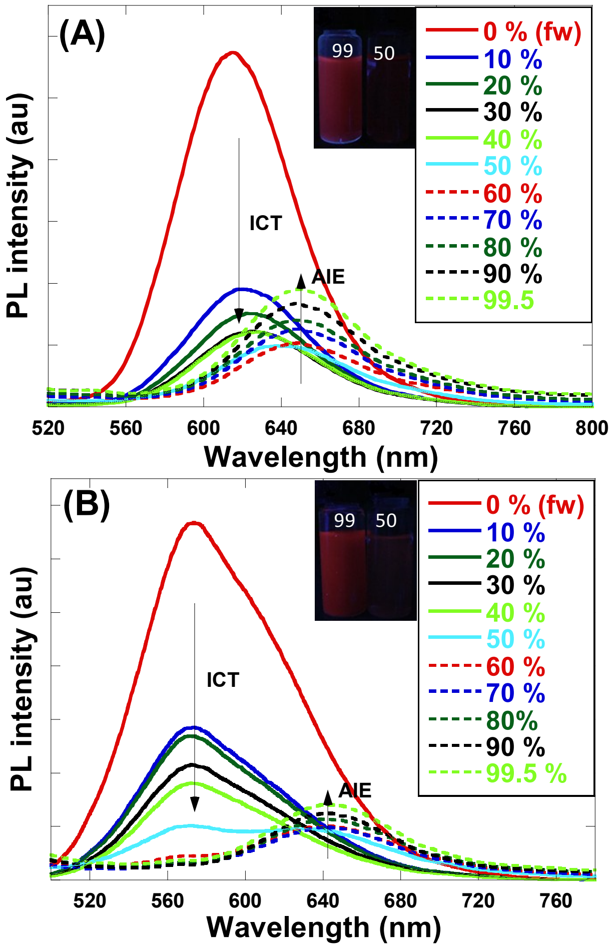

Figure S2: (A) Emission spectra of **DTPABT** in THF-water mixtures with different water fraction (f_w). Inset: photos of **DTPABT** with different water fraction under UV lamp illumination.（B）Emission spectra of **DTPEBT** in THF-water mixtures with different water fraction (f_w). Inset: photos of **DTPEBT** with different water fraction under UV lamp illumination.

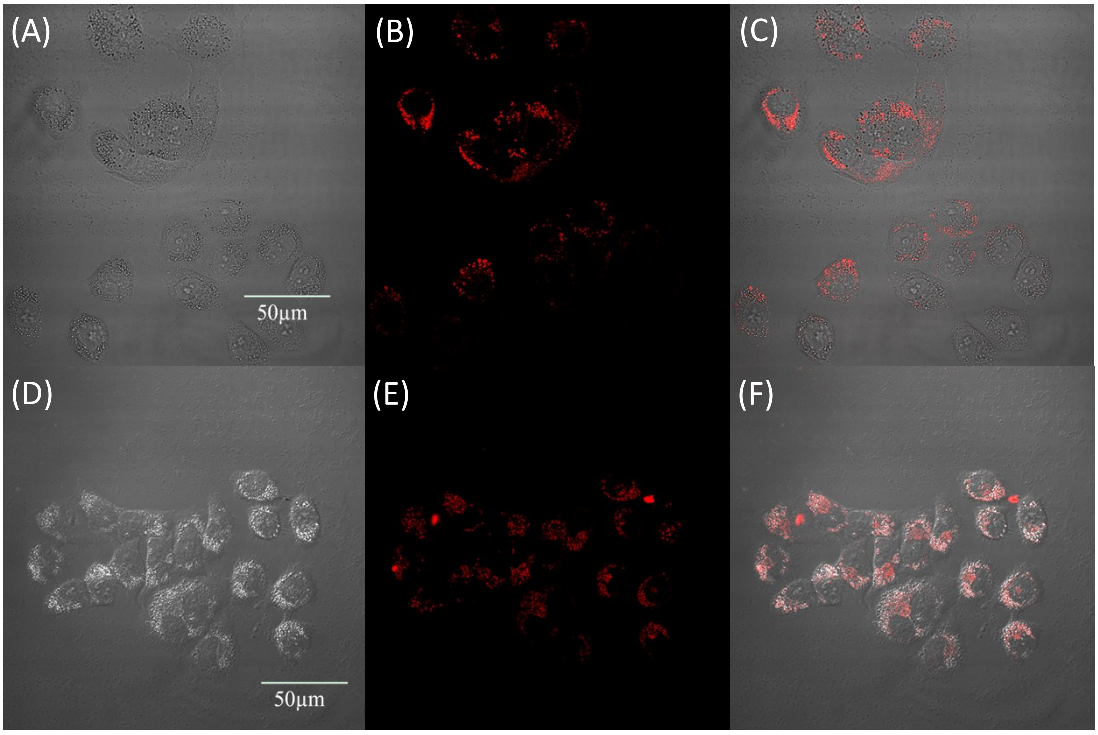

Figure S3. Fluorescence images of MCF-7 by **TPEBT** (A-C) and **DTPABT**(D-F). (A) and (D) are bright field image cells, (B) and (E) are fluorescent image, (C) and (F) are merged image, incubated for 3 hours with 20 μM.

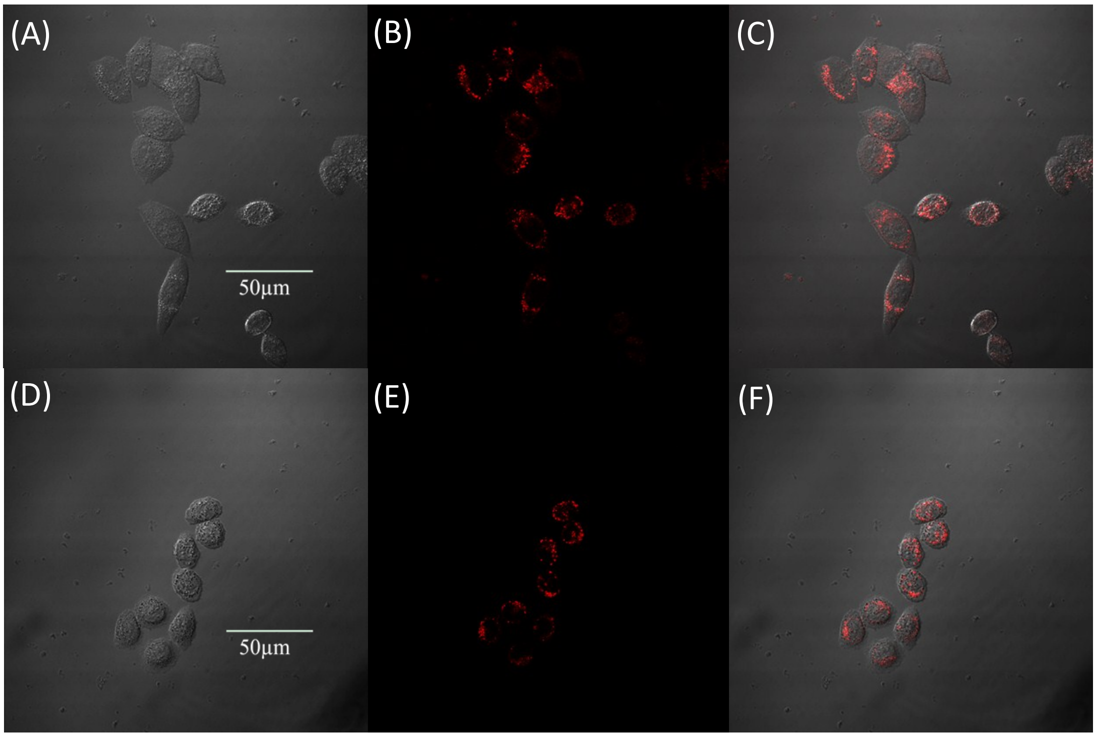

Figure S4. Fluorescence images of Hela by **TPEBT** (A-C) and **DTPABT**(D-F). (A) and (D) are bright field image cells, (B) and (E) are fluorescent image, (C) and (F) are merged image, incubated for 3 hours with 20 μM.

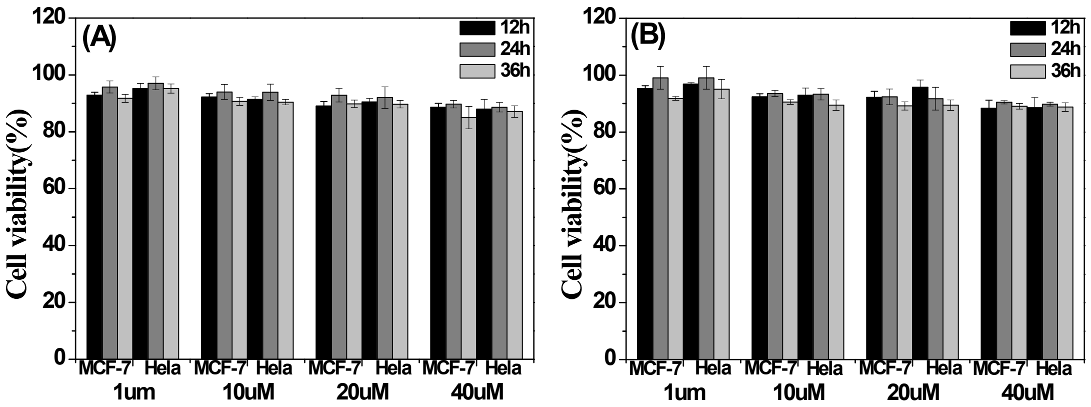

Figure S5. Metabolic viability of MCF-7 breast cancer cells and Hela cells after incubation with red emission AIE molecules (A) **DTPABT** and (B) **DTPEBT** for 12、24 and 36 h at different concentrations, respectively.

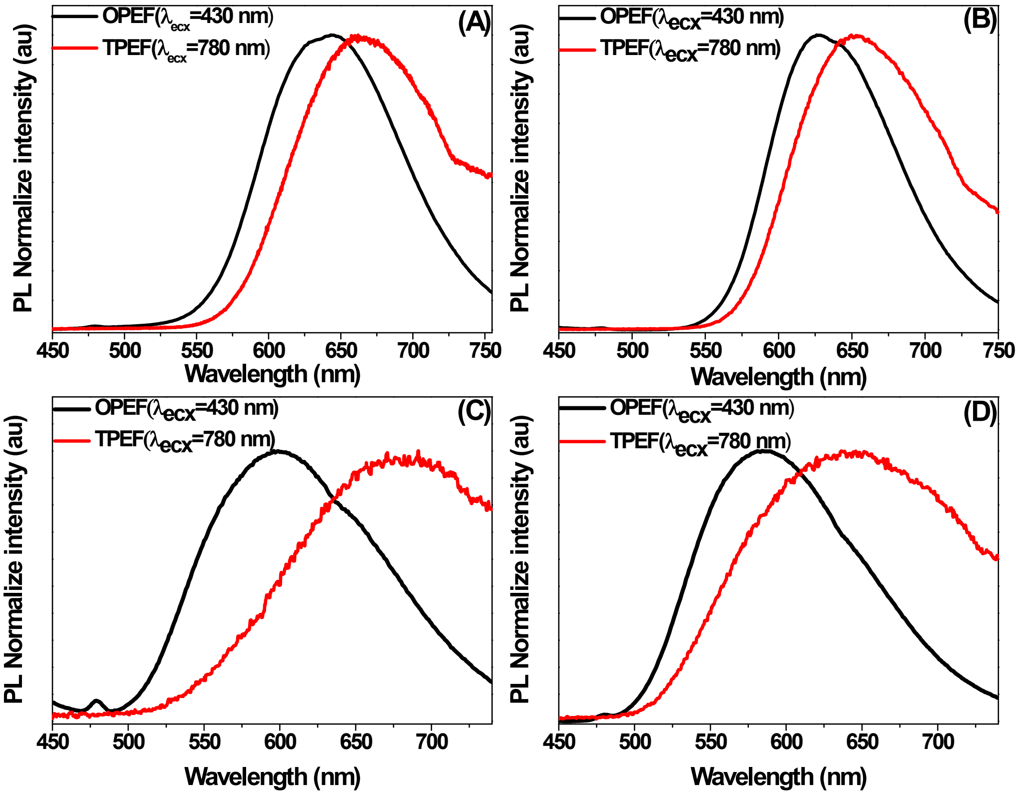

Figure S6. One-photon excited fluorescence (OPEF) and two-photon-induced fluorescence (TPEF) spectra of A) **TPABT**; B) **DTPABT**; C) **TPEBT** and D) **DTPEBT** in THF solution (10^{-5} M).

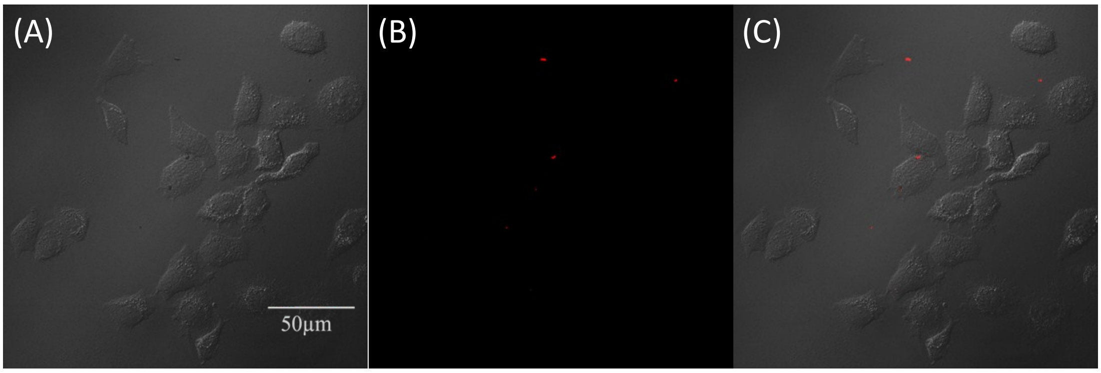

Figure S7. Two-photon excited fluorescence imaging of human breast cancer cells (MCF-7 cells) after 3 h incubation with **TPEBT** at 37 °C. The images were recorded upon 980 nm excitation with a 560–660 nm band pass filter. (A) brightfield field image cells, (B) two-photon excited fluorescence, (C) two-photon excited fluorescence/brightfield overlay. The scale bar is 50 μm.

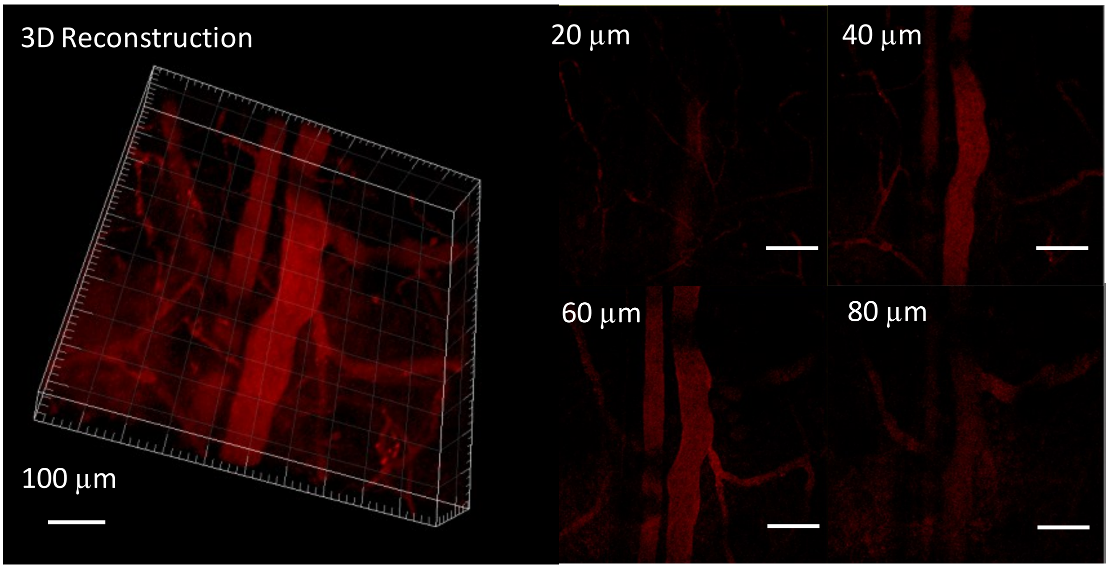

Figure S8. Two-photon fluorescence images of ear blood vessels stained with **TPEBT** NPs including 3D reconstructed images of blood vessels when **TPEBT** was excited at 980 nm, images at different vertical depths of mouse ear. All the images share the same scale bar of 100 μm.

Copies of ^1H and ^{13}C NMR Spectra

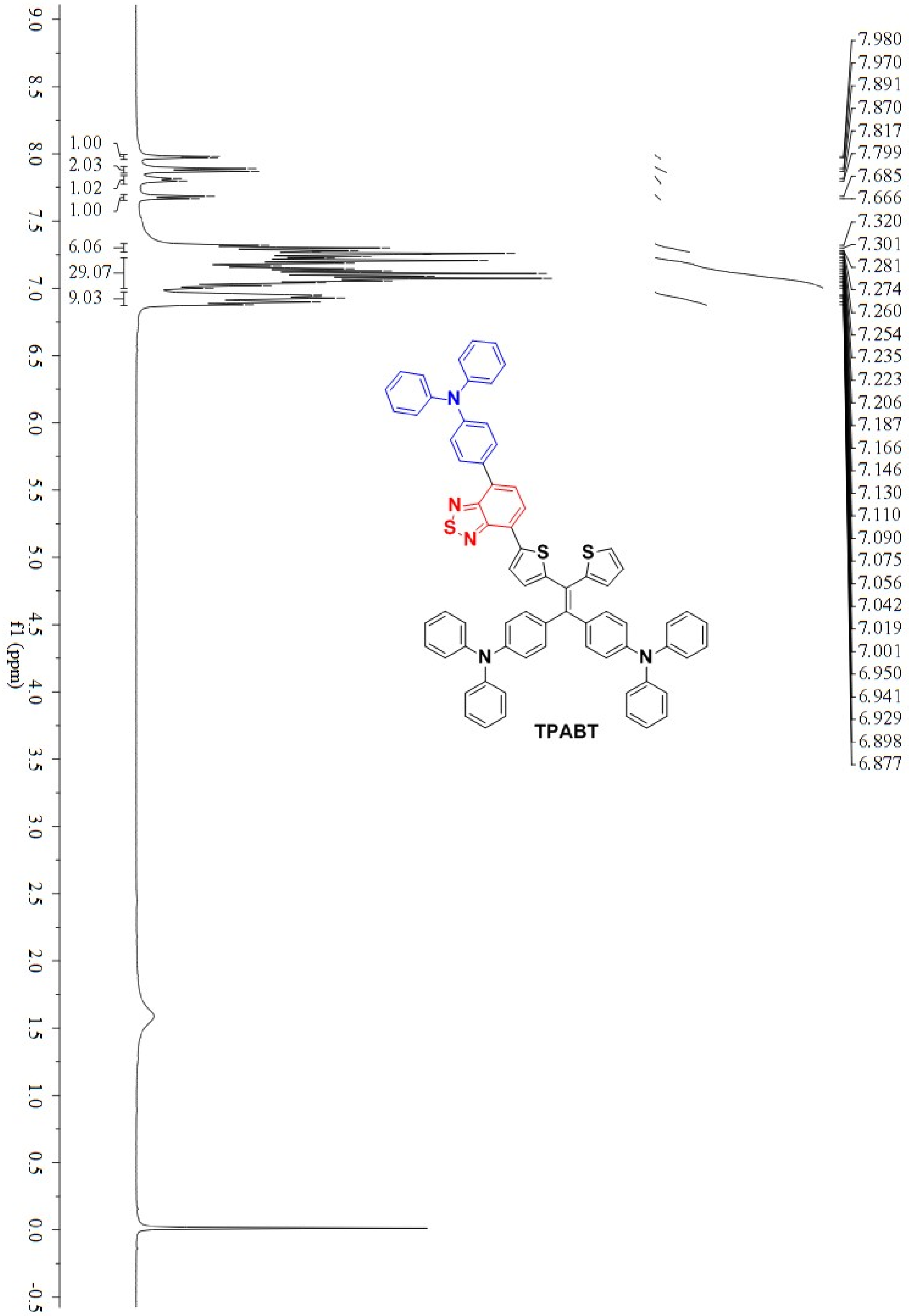

^1H NMR spectrum of **TPABT** in $CDCl_3$.

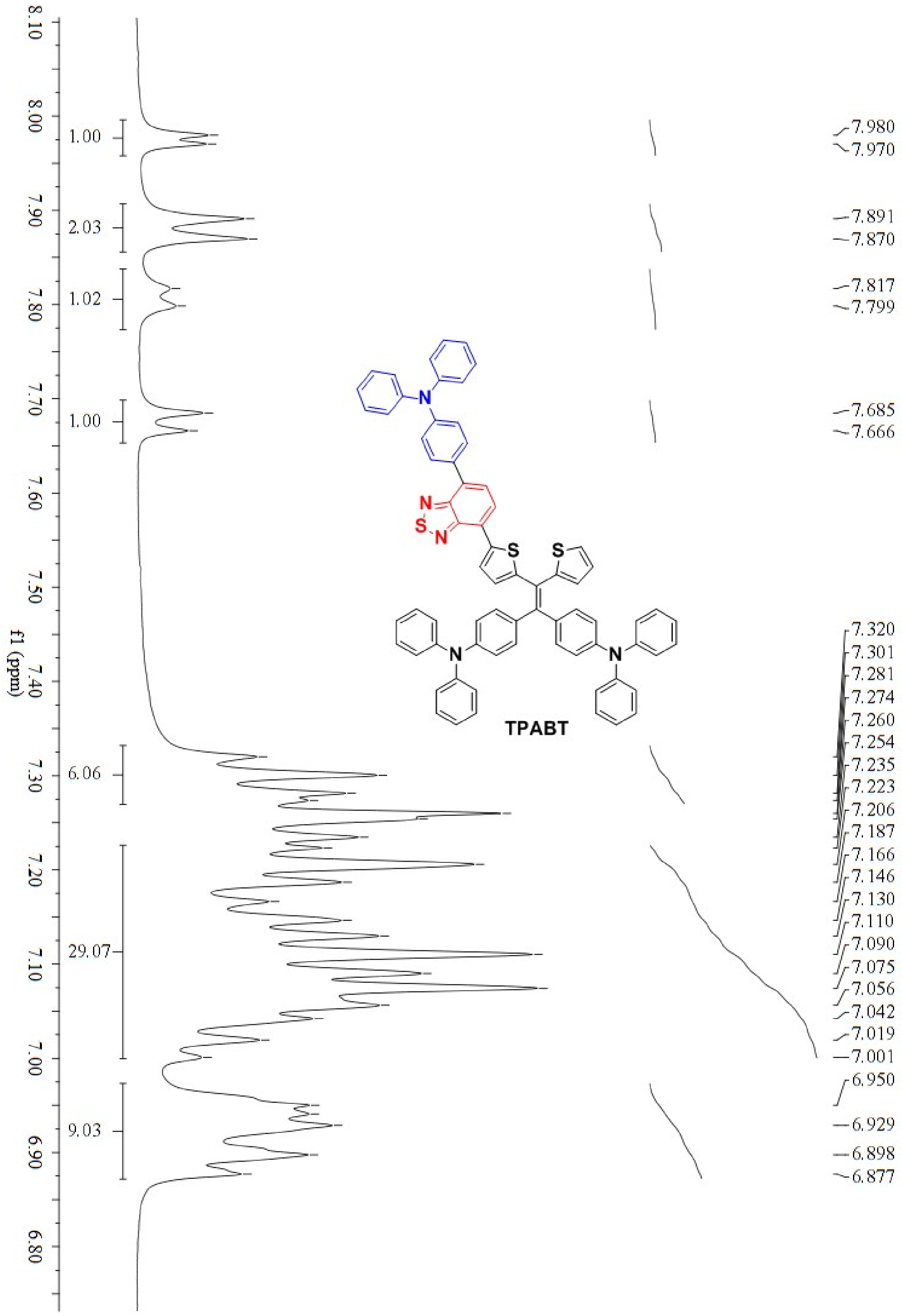

The expended ^{1}H NMR spectrum of **TPABT** in $CDCl_3$.

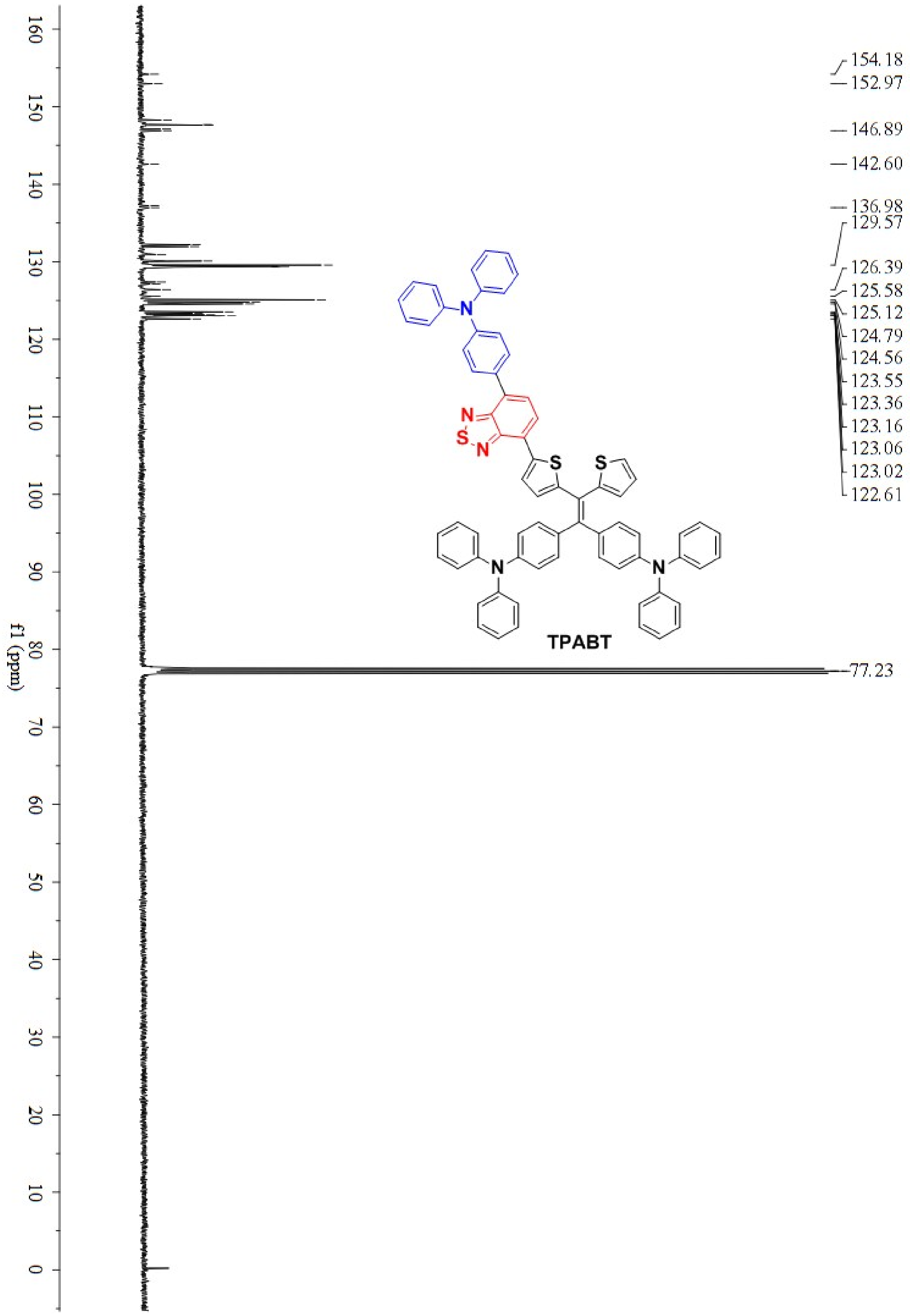

^{13}C NMR spectrum of **TPABT** in $CDCl_3$.

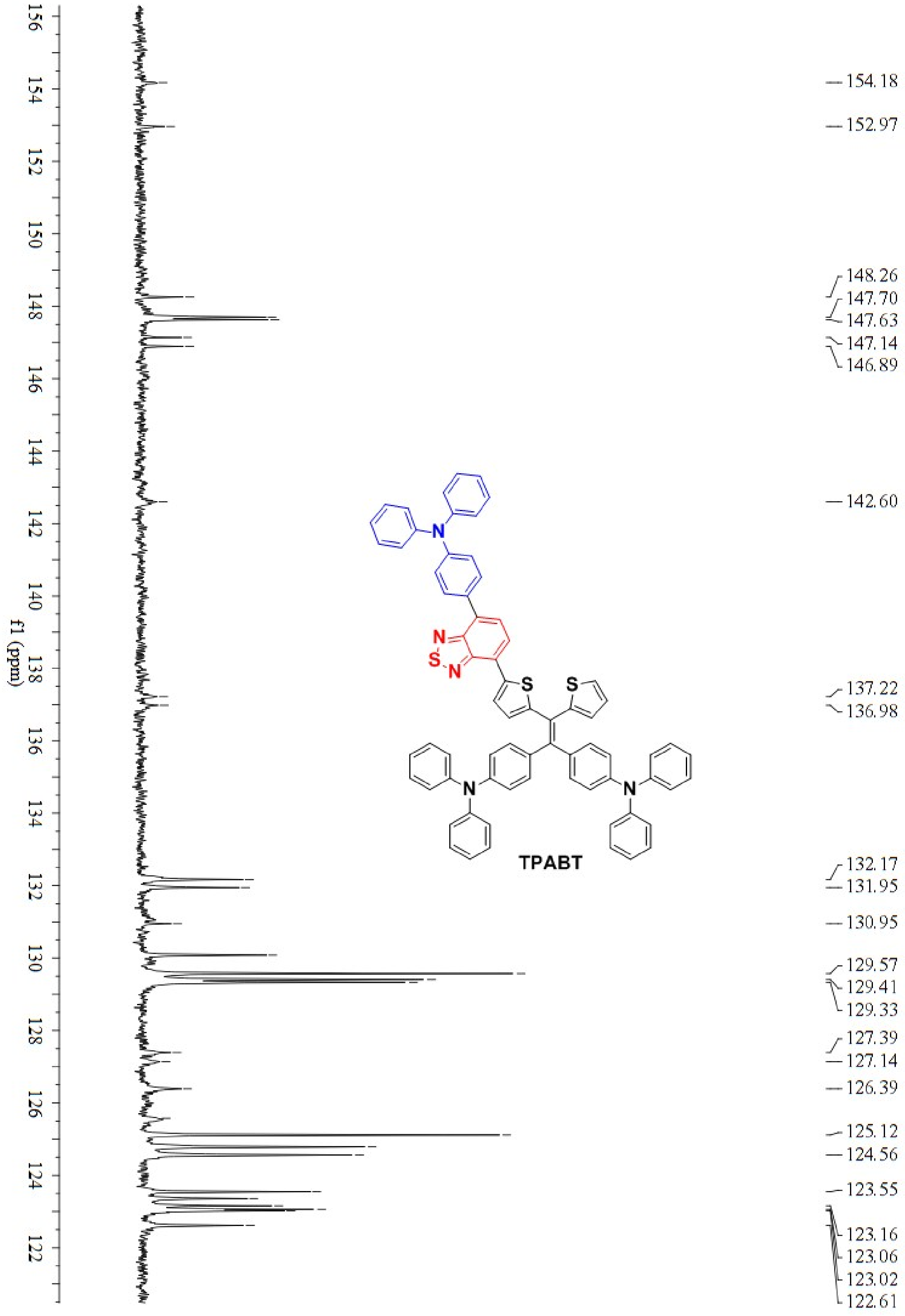

The expended ^{13}C NMR spectrum of **TPABT** in $CDCl_3$.

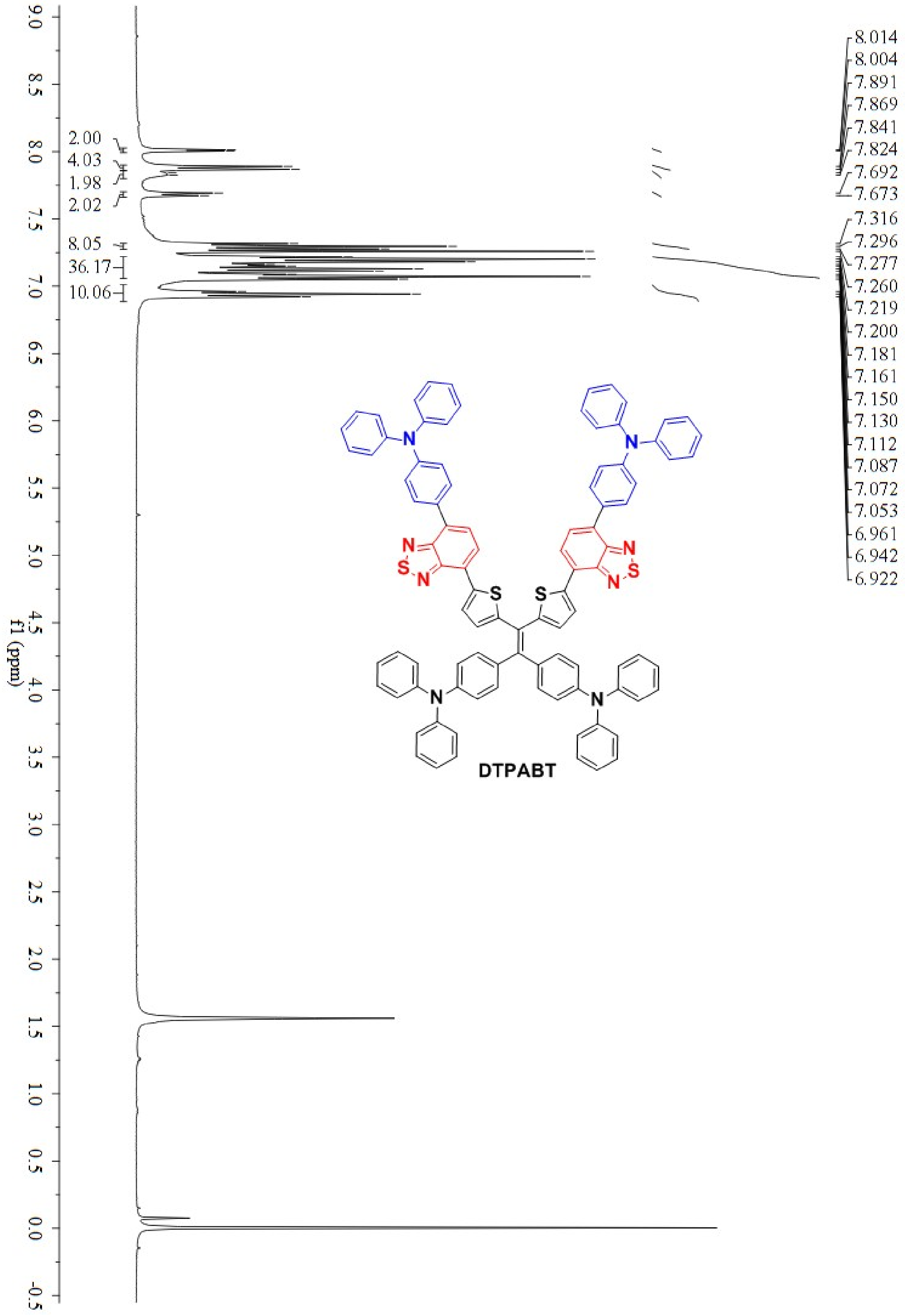

^{1}H NMR spectrum of **DTPABT** in $CDCl_3$.

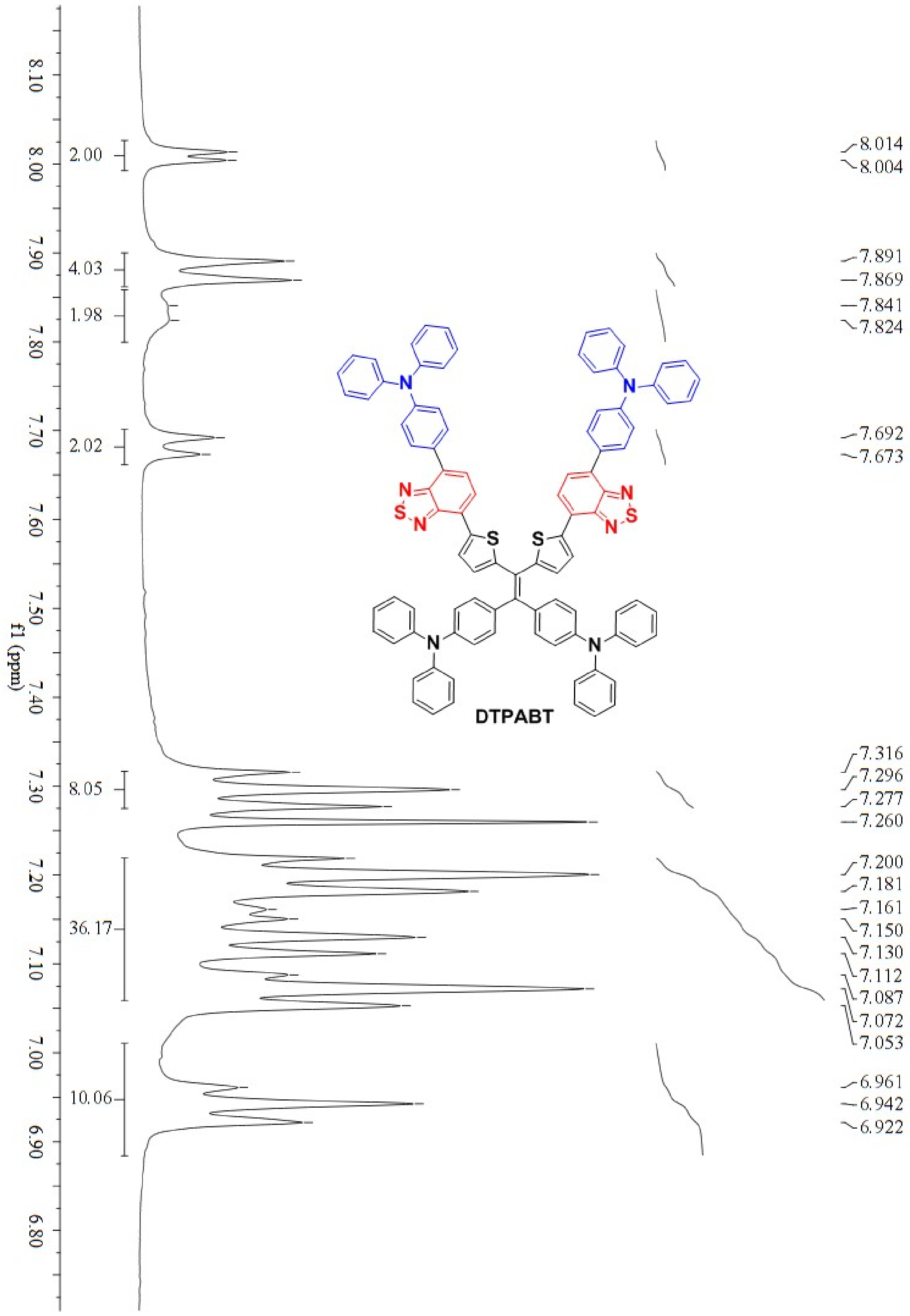

The expended ^{1}H NMR spectrum of **DTPABT** in $CDCl_3$.

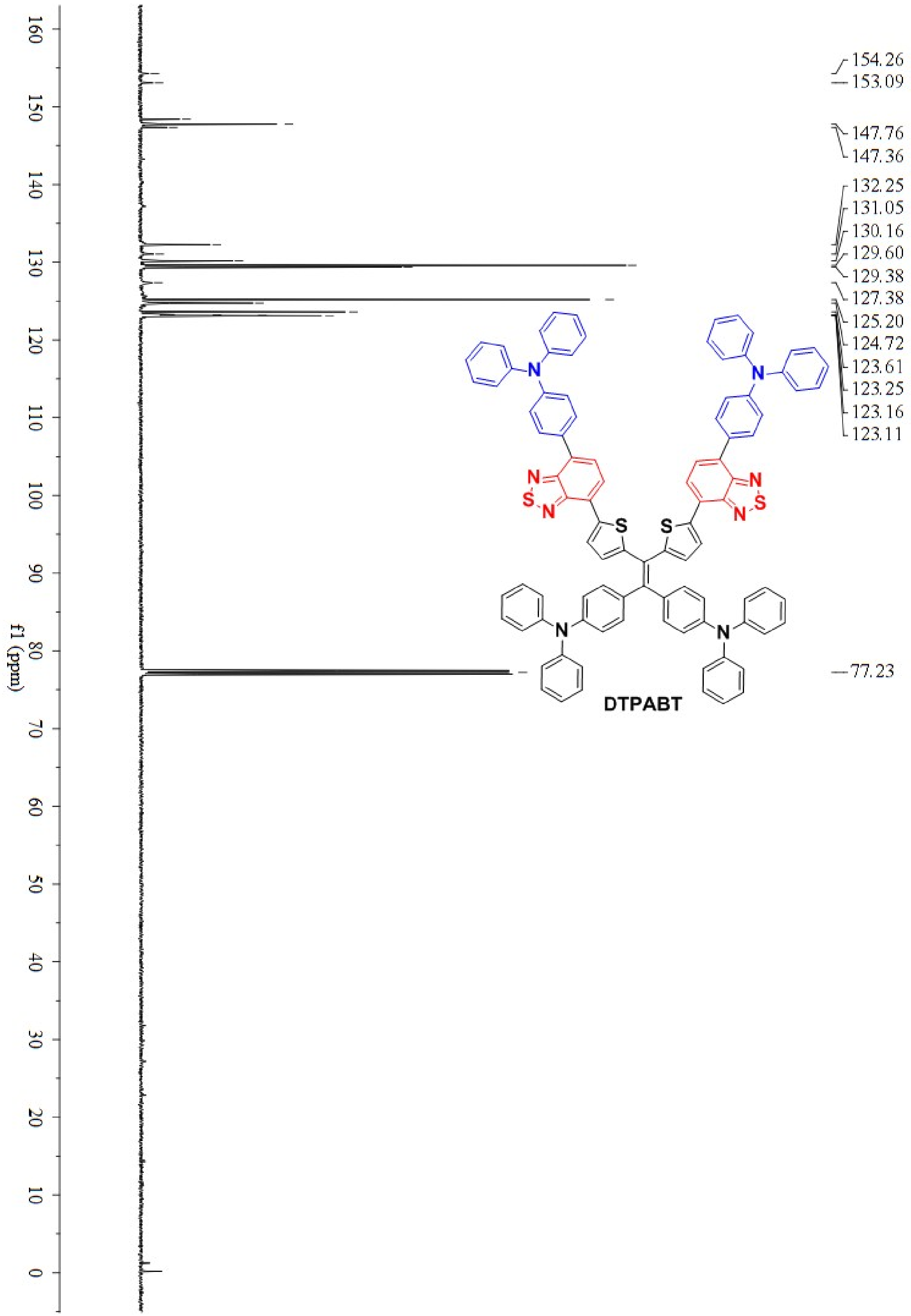

^{13}C NMR spectrum of **DTPABT** in $CDCl_3$.

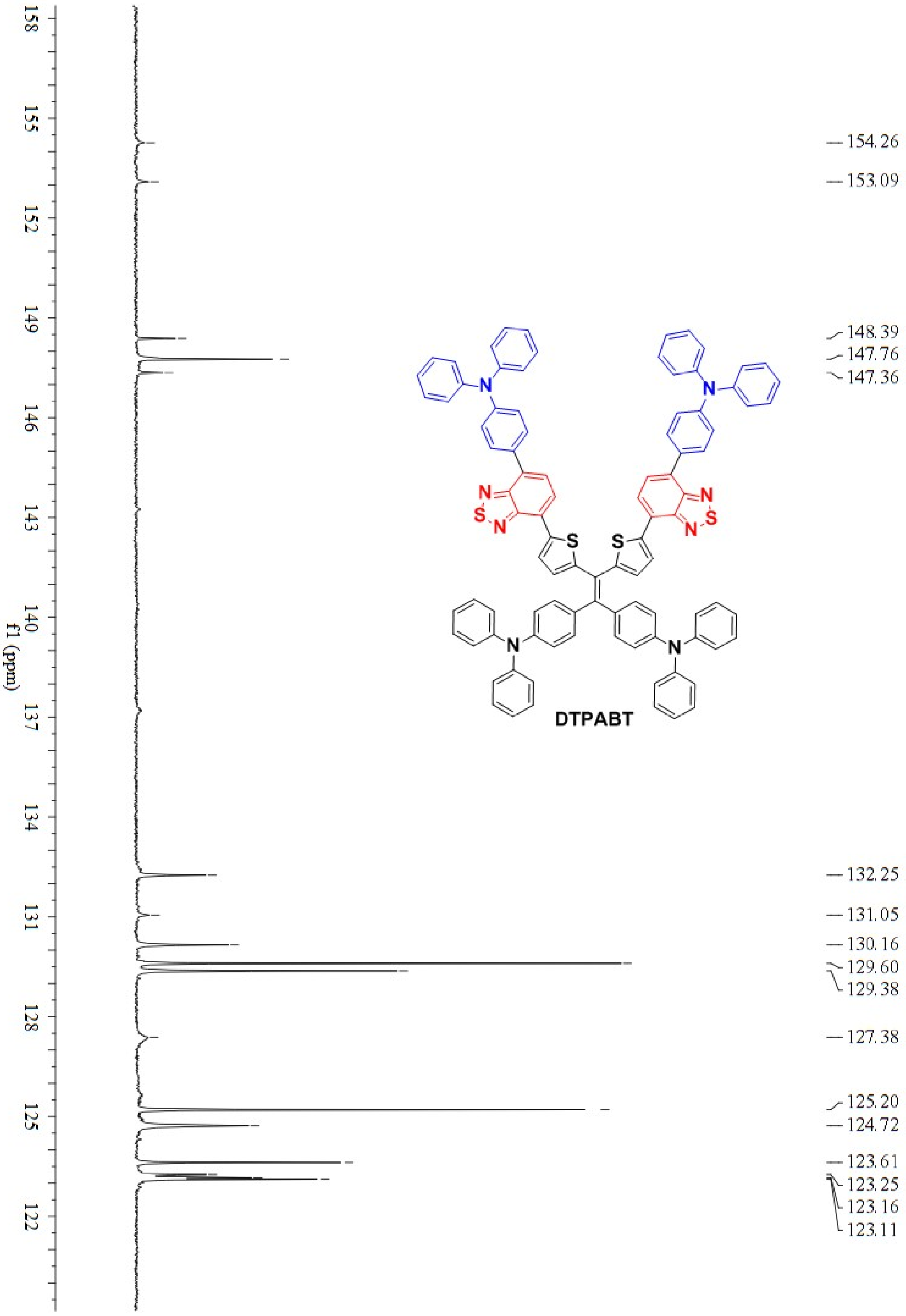

The expended ^{13}C NMR spectrum of **DTPABT** in $CDCl_3$.

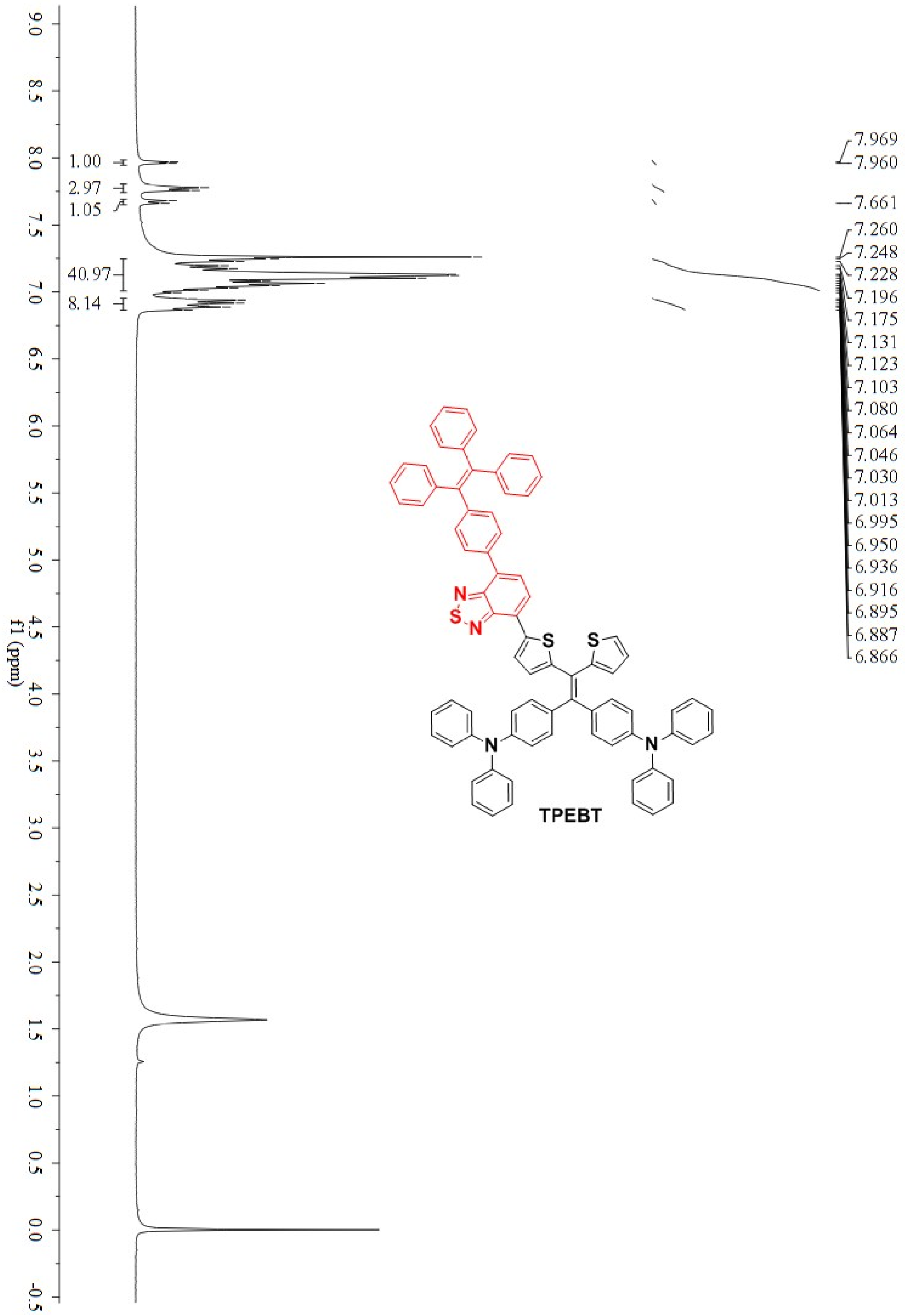

^{1}H NMR spectrum of **TPEBT** in $CDCl_3$.

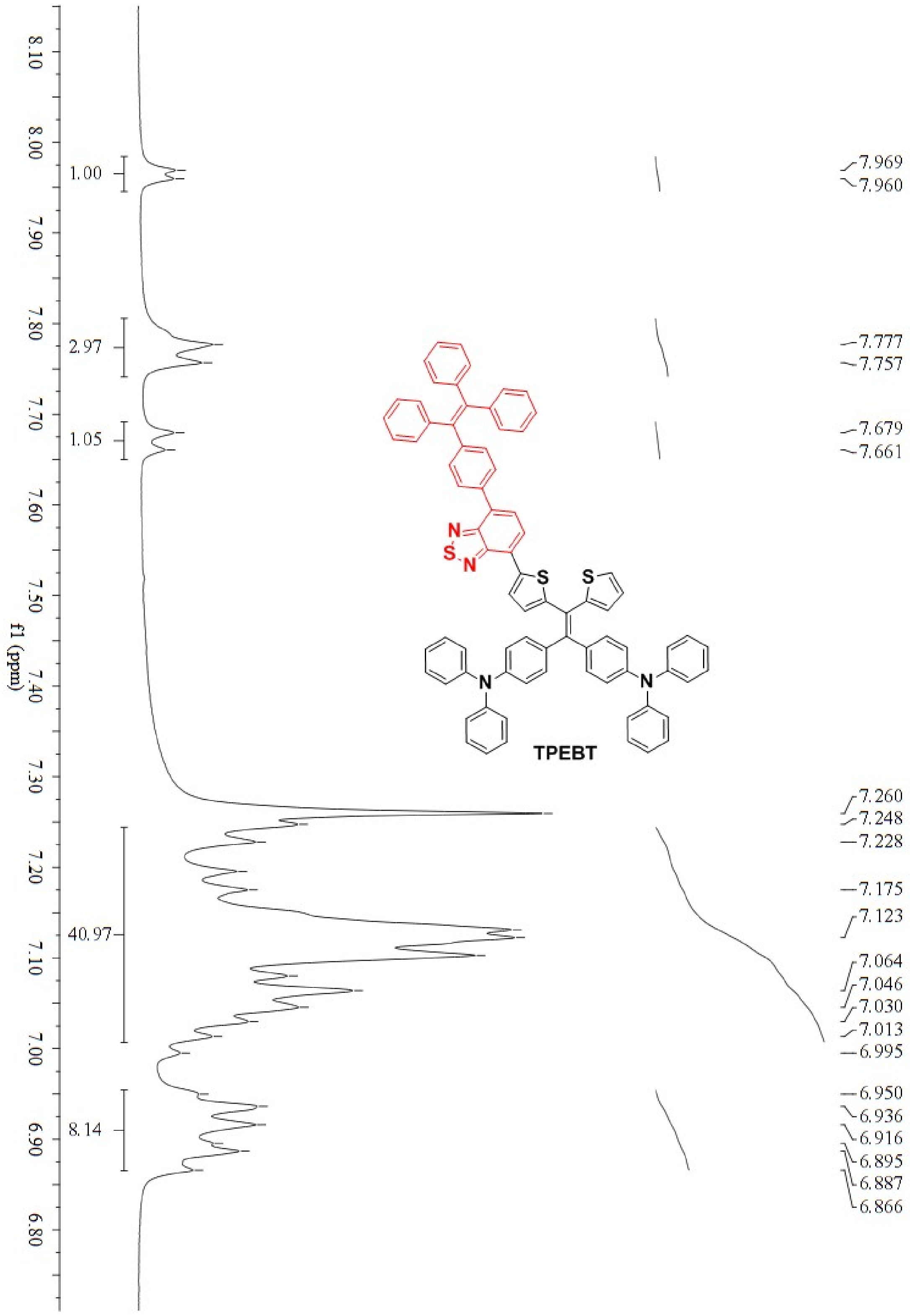

The expended ^{1}H NMR spectrum of **TPEBT** in $CDCl_3$

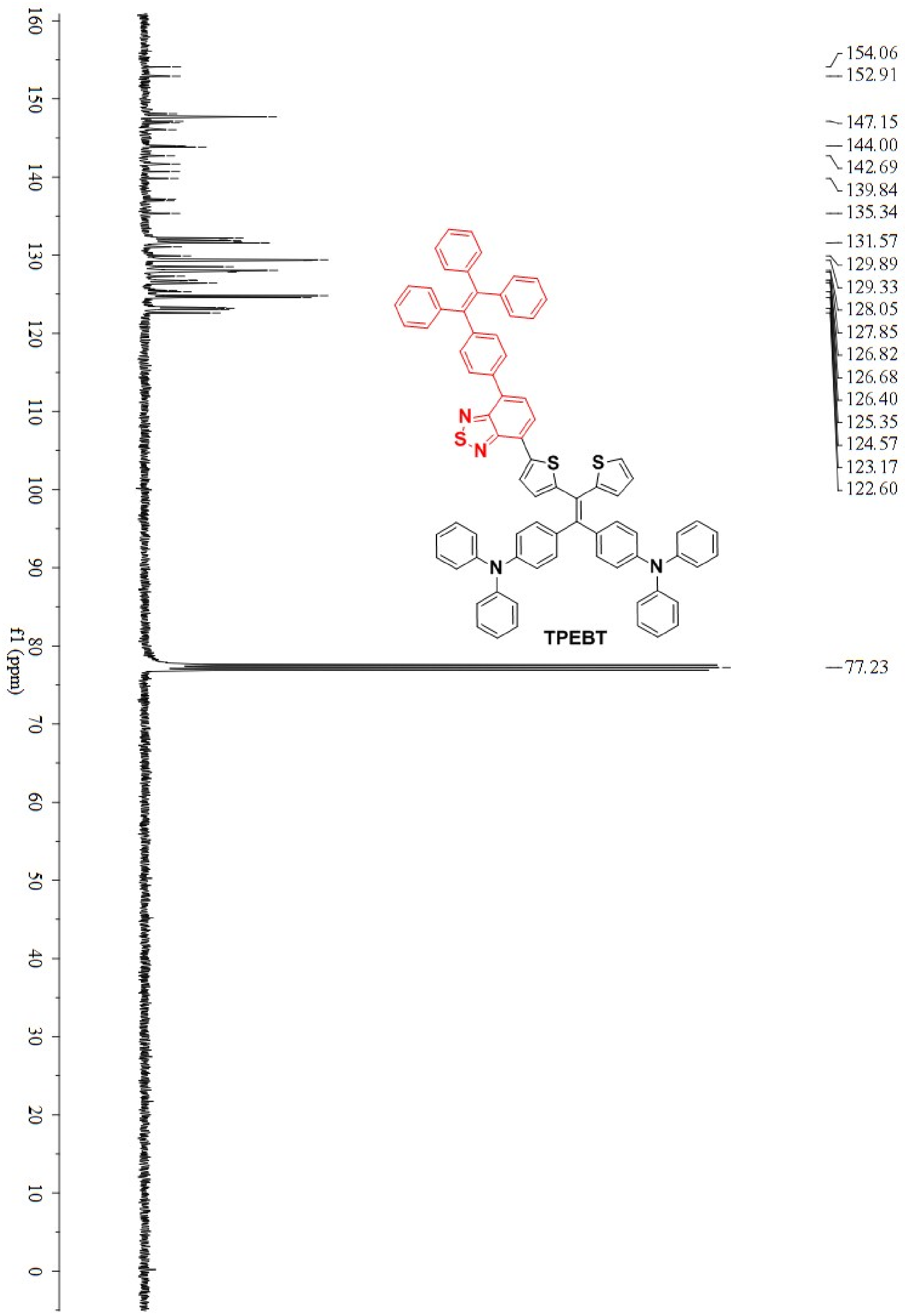

^{13}C NMR spectrum of **TPEBT** in $CDCl_3$.

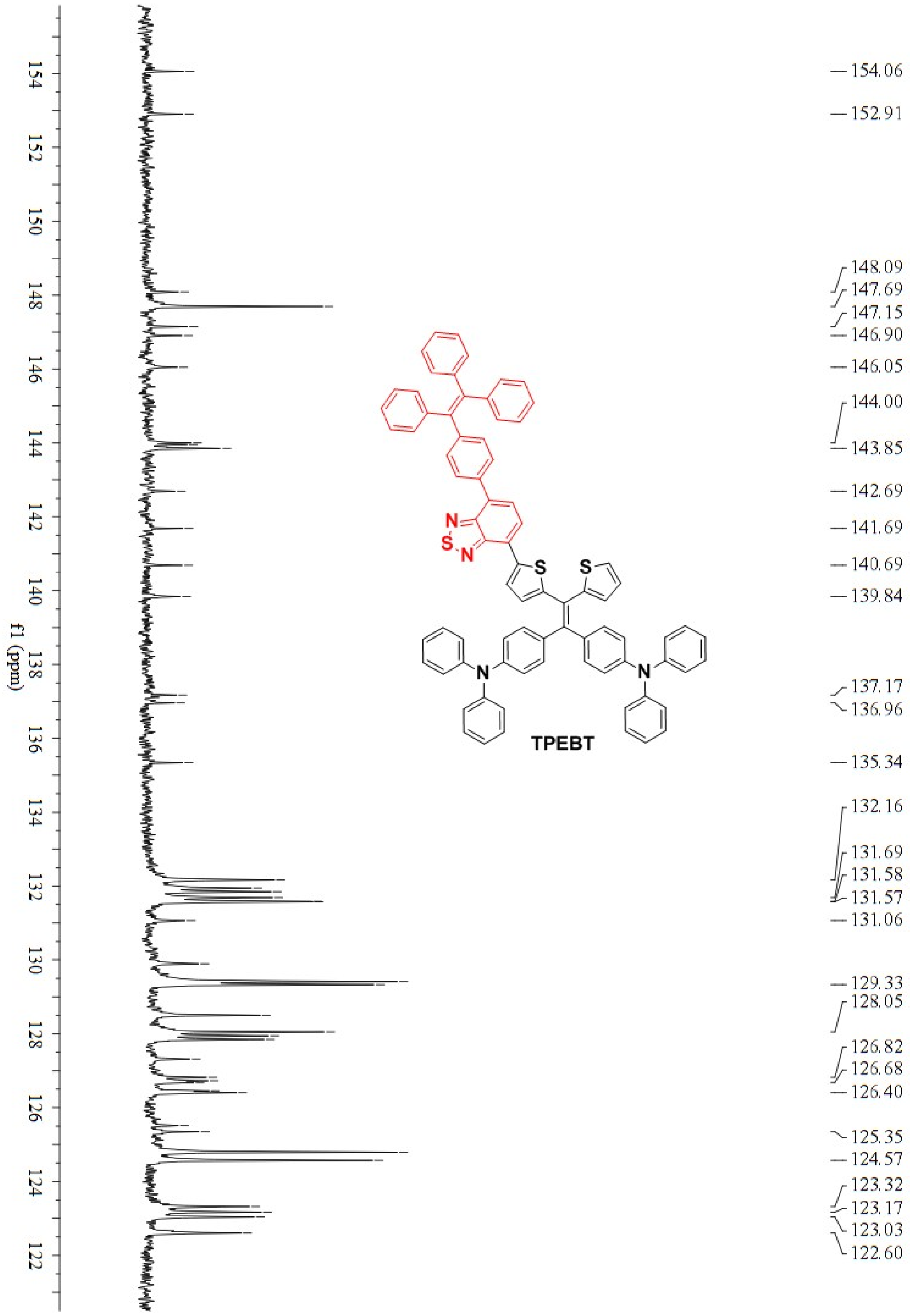

The expended ^{13}C NMR spectrum of **TPEBT** in $CDCl_3$.

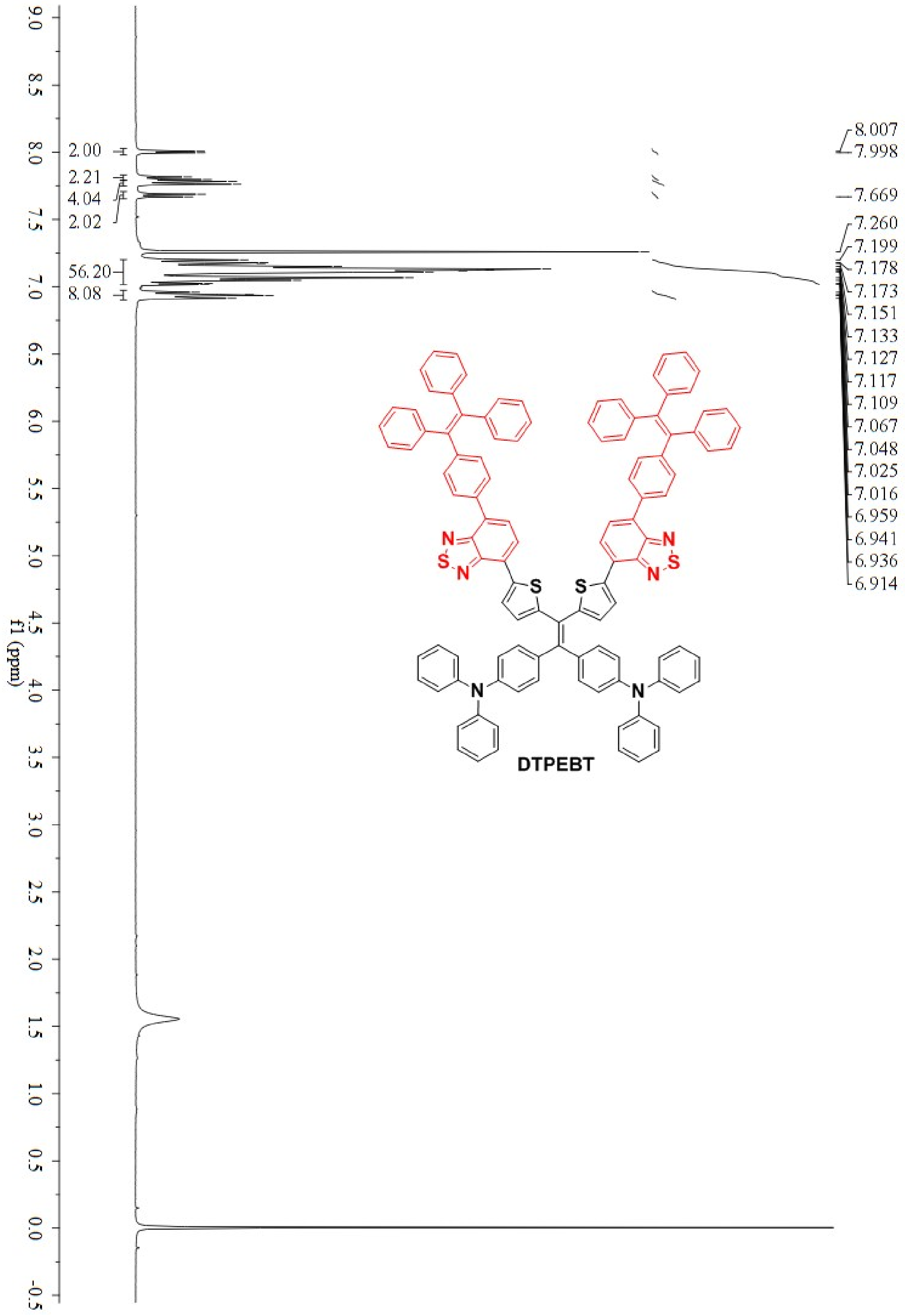

^{1}H NMR spectrum of **DTPEBT** in $CDCl_3$.

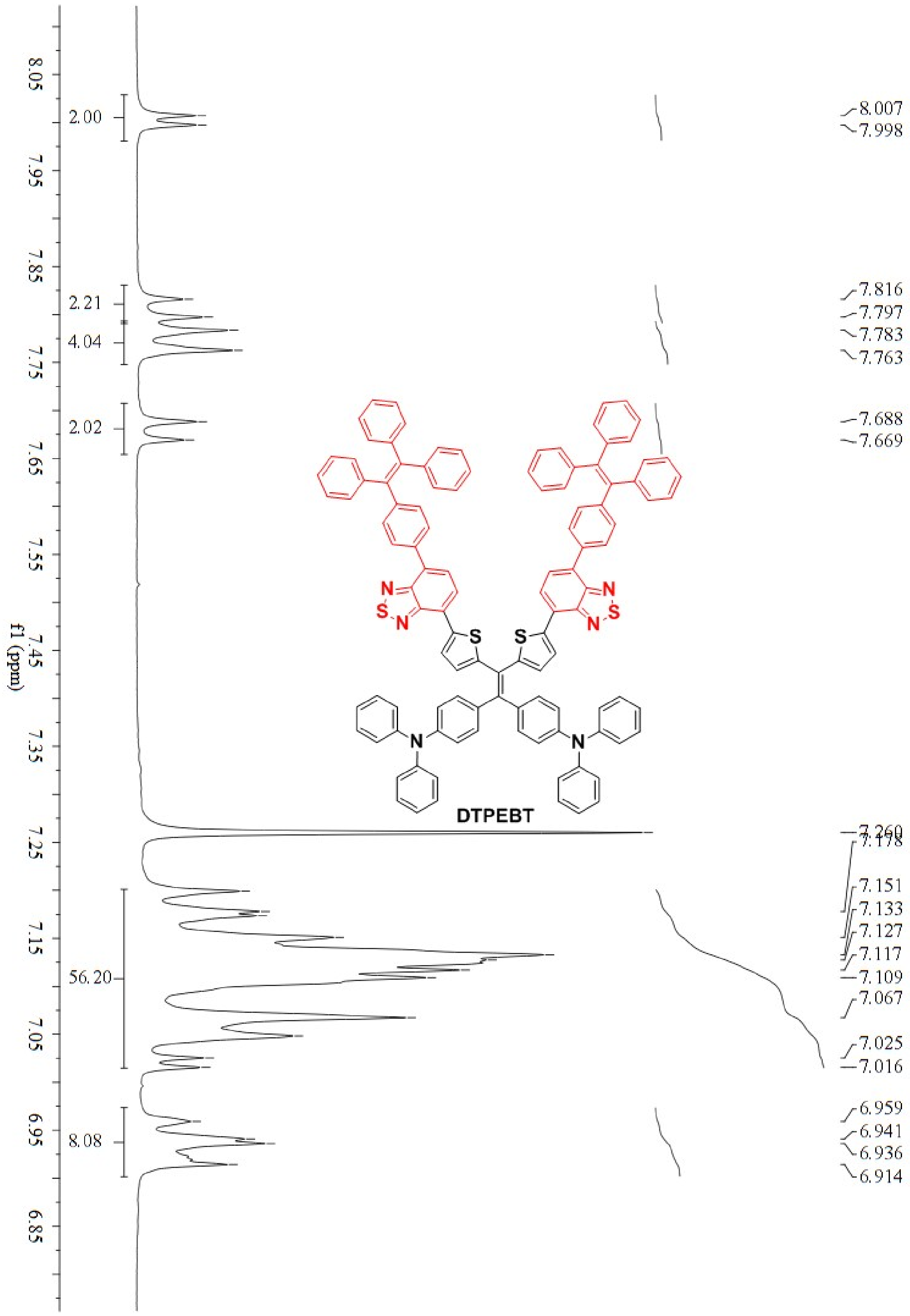

The expended ^{1}H NMR spectrum of **DTPEBT** in $CDCl_3$.

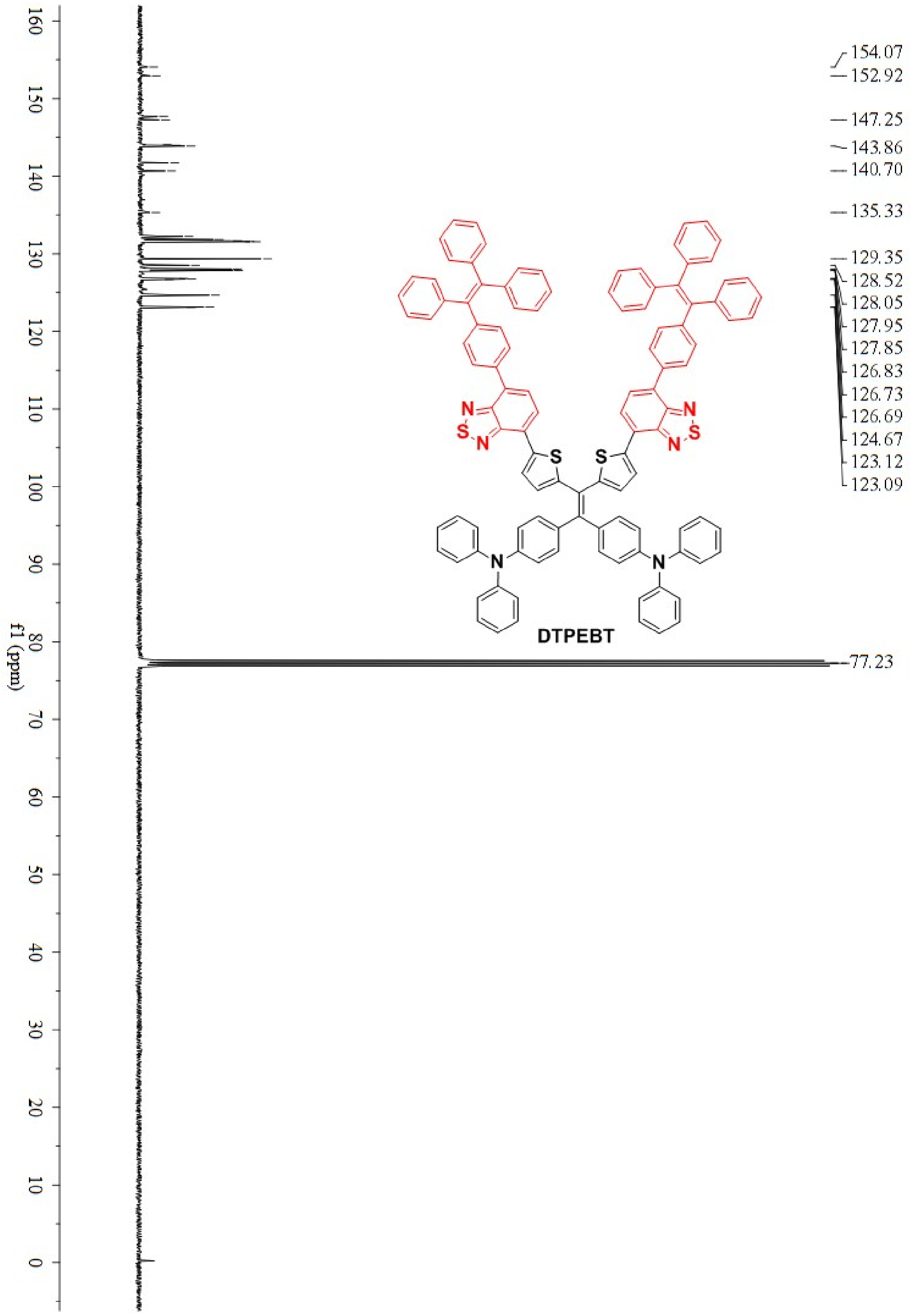

^{13}C NMR spectrum of **DTPEBT** in $CDCl_3$.

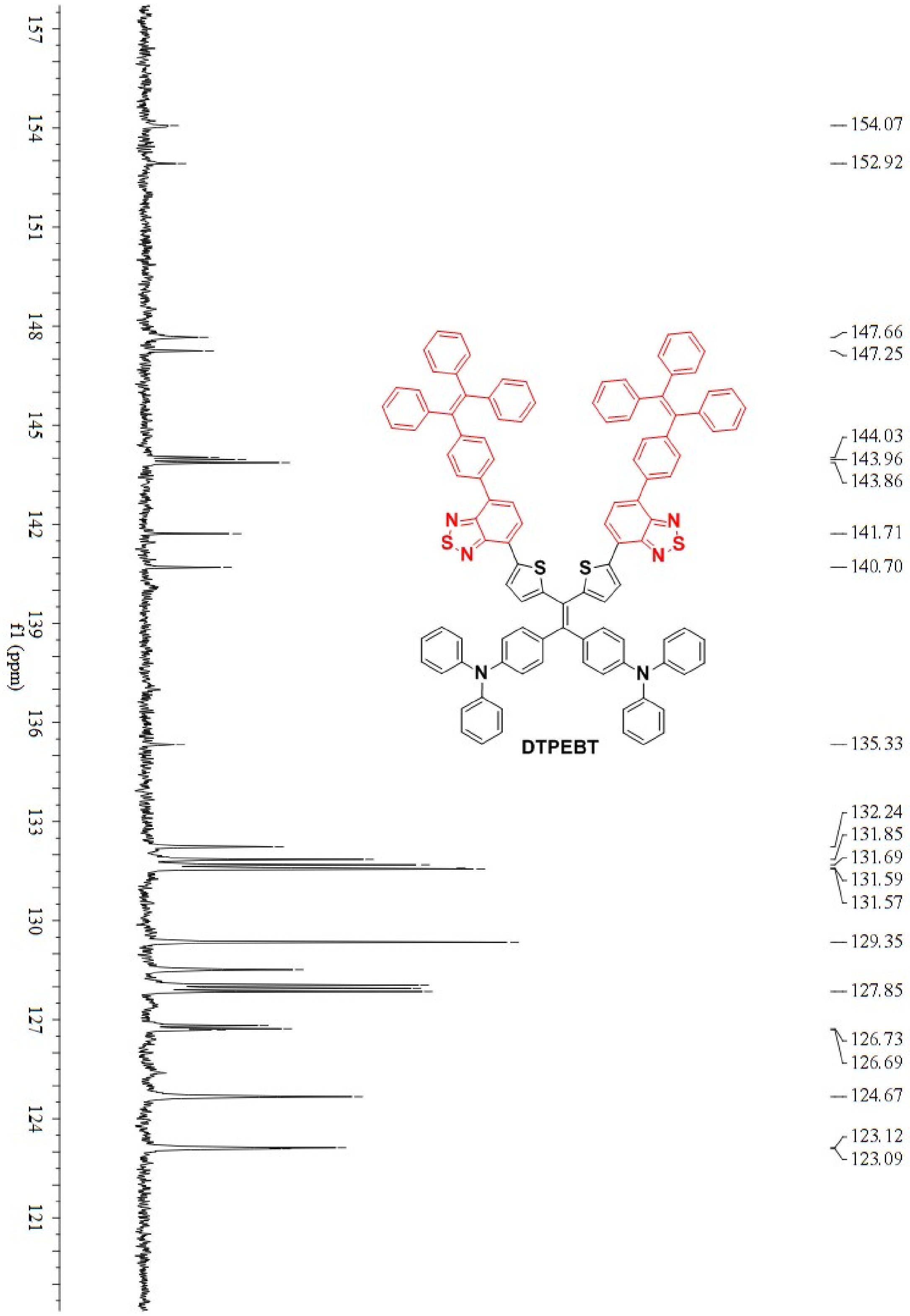

The expended ^{13}C NMR spectrum of **DTPEBT** in $CDCl_3$.
